# A Comparative Analysis of Morphological Characteristics between Endangered Local Prickly Pear and the Newly Introduced *Dactylopius opuntiae*-Resistant Species in Eastern Morocco

**DOI:** 10.1155/2024/7939465

**Published:** 2024-02-09

**Authors:** Ahmed Marhri, Mehdi Boumediene, Aziz Tikent, Reda Melhaoui, Kawtar Jdaini, Aatika Mihamou, Hana Serghini-Caid, Ahmed Elamrani, Christophe Hano, Malika Abid, Mohamed Addi

**Affiliations:** ^1^Laboratory for Agricultural Productions Improvement, Biotechnology and Environment (LAPABE), Faculty of Sciences, University Mohammed First, BP-717, Oujda 60000, Morocco; ^2^Institut de Chimie Organique et Analytique, Université d'Orléans-CNRS, UMR 7311 BP 6759, CEDEX 2, Orléans 45067, France

## Abstract

Prickly pear serves as a significant source of income for farmers worldwide, with production taking place in temperate, subtropical, and cold regions. The objective of the present investigation is to explore the morphological parameters of *Opuntia robusta* and *Opuntia dillenii* which are resistant to the white cochineal (*Dactylopius opuntiae*), as well as the local prickly pear that is currently threatened with extinction. This investigation aims to evaluate the feasibility of replacing the endangered local prickly pear with the recently introduced species *O. robusta* and *O. dillenii*. This analysis is based on a comprehensive assessment of 26 qualitative and 25 quantitative traits pertaining to cladodes and fruits. In terms of species differentiation and the selection of discriminative features, this study demonstrates the effectiveness of various statistical methods, as well as the analysis carried out according to the descriptors recommended by the International Union for the Protection of New Varieties of Plants (UPOV). Of the 51 parameters evaluated, 13 qualitative and 23 quantitative characters are significant in differentiating the species under study. This underscores the importance of quantitative traits in distinguishing different prickly pear species. Furthermore, color is identified as a crucial characteristic for discriminating between the studied samples. *O. robusta* is characterized by its high fruit weight, large size, greater pulp content, and high pulp-to-peel ratio, all of which are desirable traits for fresh consumption. Additionally, *O. robusta* has the highest number of fully developed seeds, making it an attractive option for use in the cosmetic industry. This characteristic renders the *O. robusta* a potential substitute for the endangered ecotype . However, *O. robusta* is distinguished by its short stalk, which poses a challenge for fruit harvesting and leaves it susceptible to physical damage and quality loss. Conversely, *O. dillenii* displays a low pulp content, which serves as a critical indicator of fruit quality. The only desirable agronomic trait of this species is its elevated seed content, which has the potential for utilization in oil production for the cosmetic industry.

## 1. Introduction

The prickly pear, which belongs to the Cactaceae family, stands as a preeminent plant with nearly 130 genera and around 2000 species, according to Bouzroud et al. [1]. The nopal cactus, native to Central America, is widely spread throughout the continent, with Mexico having the largest cultivated area of approximately 70.000 hectares, as reported in [2]. The cultivated area is distributed among 15.000 hectares for fodder production, 10.500 hectares for cactus pear production, and 51.112 hectares for commercial fruit production, as highlighted in [3]. In 1552, Christopher Columbus introduced the prickly pear cactus to Europe during his first expedition to America. Subsequently, in the 16th century, the plant was further disseminated to the Mediterranean region following Spanish expansion and the return of Arabs to their countries in 1610 [4]. Mexico holds the leading position as the primary source and major producer of prickly pear fruit, while Italy holds the second position [5].

The ecological adaptability of the nopal cactus, coupled with its highly efficient and easily implementable vegetative propagation, distinguishes this plant species from others. These features have enabled nopal cactus to thrive in various regions worldwide, including Mediterranean areas as well as arid and semiarid regions, where water scarcity poses a significant limitation to the reproduction of most plants. Additionally, the plant's drought resistance ability enables it to attain high yields even in stressful environments, making it an ideal crop for the development of marginal lands and a crucial plant in the mitigation of desertification and soil erosion. Cactus pear presents a promising crop with immense potential for breeders, farmers, and food technologists [6]. Given the persistent problem of climate change, global desertification, and declining water resources, the utilization of *Opuntia* sp as a food production system that encompasses both fruit and vegetable parts becomes increasingly crucial [7]. Furthermore, the nopal cactus stands out as a valuable resource not only in the agricultural and food production sectors but also in the cosmetic and pharmaceutical fields. Its diverse applications and benefits make it a plant of significant interest to researchers and industry professionals alike [8].

In the eastern region of Morocco, the prickly pear serves primarily for delineating plots and as a natural fence around homes. The plant's resilience in challenging environments, such as valleys and mountains, makes it a preferred choice for cultivating fruit crops in these areas. The Moroccan population, along with several other countries globally, highly values the fruit of this species, contributing to its increased profitability.

As a result of these characteristics, the cultivation of this plant has experienced a consistent increase, with the area of cultivation expanding from around 60.000 hectares in the early 2000s to over 150.000 hectares in 2017 [9]. Unfortunately, the rapid spread of the white cochineal (*D. opuntiae*), which was introduced to Morocco in 2015 [10], has resulted in widespread destruction in almost all regions of the eastern part of the country, endangering the survival of the local prickly pear. Consequently, farmers have begun importing resistant species to mitigate this issue. Currently, two new resistant species, *O. robusta* and *O. dillenii*, have been successfully introduced in the northeastern region of Morocco. These species are among the eight cochineal scale insect's resistant species that the National Institute of Agronomic Research (INRA) has included in the official catalog for use in future plantings and site restoration following devastation by cochineal scale insects.

The performance of different species depends on their adaptability to agroecological diversity [11]. Hence, it is imperative to ensure that the newly introduced resistant cultivars can adjust to the climatic conditions prevalent in the northeastern region. This study aims to achieve two primary objectives: firstly, to compare the morphological characteristics of the local prickly pear, which is at risk of extinction, with the two species that are resistant to carmine cochineal, and secondly, to assess the effectiveness of recently introduced cultivars and pinpoint accessions exhibiting favorable agronomic characteristics, considering their current and potential applications, particularly in fruit production and oil evaluation. The research seeks to ascertain the extent to which the newly introduced resistant species can replace the local prickly pear. The study encompasses an analysis of both the cladodes and fruits of the prickly pear.

## 2. Materials and Methods

### 2.1. Research Methodology

The International Union for the Protection of New Varieties of Plants (UPOV) guidelines [12] were followed for analysing cladodes and fruits of the different studied species. The study also took into account the General Introduction and related TGP (Development of Test Guidelines) documents.

### 2.2. Study Location

**Figure 1:**
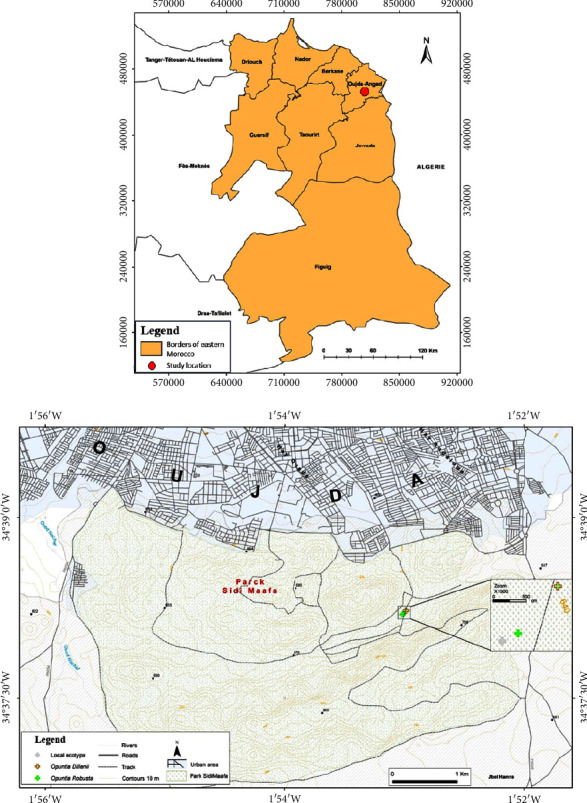
Geographic distribution of the studied species: *Opuntia robusta*, *Opuntia dillenii,* and local ecotype.

**Figure 2:**
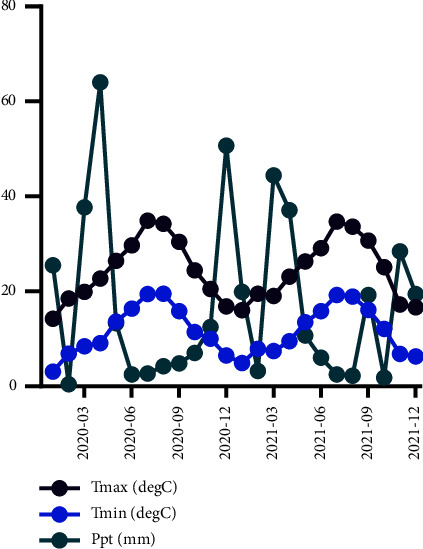
Climatic conditions, including monthly average weather, temperature, and precipitation of the Oujda region during the two consecutive years of study (2020-2021).

Three populations of prickly pear were selected from the province of Oujda located in northeastern Morocco for analysis (Figure 1). This region has an elevation of 550 meters above sea level and experiences a Mediterranean climate with cold winters and hot summers (Supplemental Table 1). Precipitation in the region is irregularly distributed over time, as depicted in Figure 2.

In accordance with information obtained from (climate.northwestknowledge.net), the warmest month in the Oujda region of northeastern Morocco is July, with an average temperature of 34.8°C, while January is the coldest month, with an average temperature of 4°C (Figure 2).

Data collection for all samples occurred over the course of two consecutive years (2021-2022), and 38 morphological characteristics pertaining to cladodes and fruits were evaluated to describe the three genotypes, as presented in Table 1.

### 2.3. Biological Material

**Figure 3:**
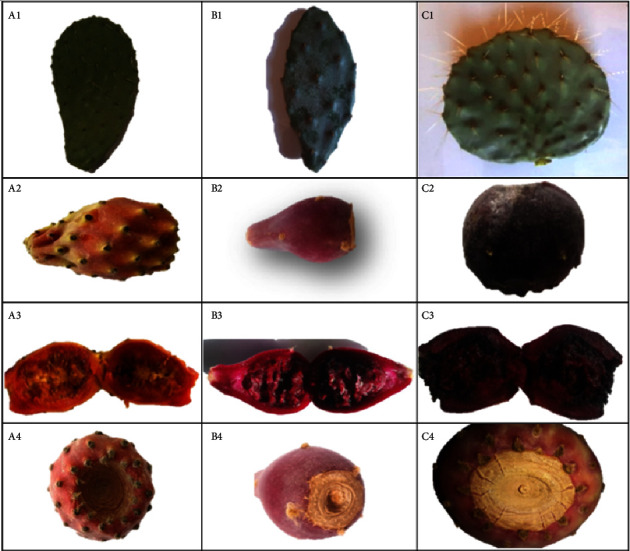
Morphological variability of cladodes and fruits of local prickly pear (A), *Opuntia dillenii* (B), and *Opuntia robusta* (C). Cladodes diversity: A1-B1-C1. Fruit diversity: A2-B2-C2. Longitudinal sections of the fruit: A3-B3-C3. Receptacle scars of the fruits: A4-B4-C4.

The methodology employed in this study involved collecting two parts from each of five healthy plants that were more than three years old and had at least three successive cladodes. The sampling was carried out during summer of the two consecutive years 2020 and 2021. The observations of the fruits were conducted on 20 samples that were processed within 24 hours (Figure 3). The samples were weighed before peeling, and the edible part (pulp) of each fruit was crushed and strained to separate the seeds from the remaining pulp. The seeds were then subjected to shade drying to eliminate surface moisture. Weight measurements of both fruits (mature fruits) and cladodes (young cladodes) were obtained using a precise balance (0.01 g) manufactured by Statorius AG in Gottingen, Germany. Additionally, the dimensions of the fruits, including internal, external, and depth measurements, were obtained using a caliper.

## 3. Data Analysis

In order to examine the relationships among the different accessions under study, a variety of statistical procedures were employed. Regarding the assessment of normal distribution, normality is considered acceptable if the calculated *p* value of the Shapiro–Wilk test surpasses the alpha threshold (5%). On the other hand, if the *p* value from the Levene test exceeds 5%, the null hypothesis is accepted, indicating the homogeneity of variances (Levene statistic 0.03; *p* > 0.05). The response variables did not meet the assumptions of normal error distribution and homogeneity of variances; therefore, the data were transformed into rank [13] and the analysis was carried out so as not to violate the assumptions. Statistical analyses were carried out using the IBM SPSS Statistics 25 software. Only variables with a confidence level higher than 95% were considered significant. The T2 de Tamhane post hoc test was then used to carry out multiple comparisons of the mean values.

For the quantitative traits, principal component analysis (PCA) which is dimension reduction method was used to represent a set of observed variables in terms of a smaller number of variables, which were then employed for K-means cluster analysis in conjunction with ANOVA to assign cluster membership to individuals. To evaluate the relationships between various qualitative descriptors, the MCA method was used, with the mean Cronbach's alpha index value being considered acceptable if it exceeded the minimum threshold of 0.70 [14].

Color measurement was assessed using the method described in [15] utilizing a chromameter C-410 and the CIE lab system. The color measurements were calculated using the following formulas.

Saturation metric is calculated as follows:

(1) $$\begin{array}{c} C=\sqrt{\left(a^{2}+b^{2}\right)}. \end{array}$$

The tint is calculated as follows:

(2) $$\begin{array}{c} H=\text{arc tg }\left(\frac{b}{a}\right). \end{array}$$

The distance between two colors is calculated as follows:

(3) $$\begin{array}{c} \text{dE}=\sqrt{\left(dL^{2}+{\text{dC}}^{\cdot \cdot 2}+{\text{dH}}^{\cdot \cdot 2}\right)\;}. \end{array}$$

## 4. Results

Variations were noted across the samples concerning 51 parameters associated with cladodes and fruits. Notably, color emerged as one of the most crucial traits for distinguishing among the studied samples. While the fruits could be easily distinguished, the cladodes were difficult to differentiate due to their high similarity. To address this issue, a chromameter device was employed, which revealed substantial differences in color among the samples (Table 2, Table supplemental 2). *O. dillenii* displayed the higher hue value and the lowest lightness. However, the local genotype showed the highest chroma value with moderate results of each for both hue and lightness. In contrast, *O. robusta* registered the higher lightness value, along with moderate results of each of chroma and the hue.

### 4.1. Quantitative Morphological Traits Variability

To assess the morphological diversity among the studied species, separate analyses were conducted on both cladodes and fruits. One-way MANOVA, PCA, and K-means tests were employed based on 25 quantitative parameters for this purpose. One-way MANOVA test conducted on the fruits revealed statistically significant differences among the studied species (*p* < 0.05). The subsequent post hoc test, enabling pairwise comparisons of groups, indicated significant differences between the examined groups (Table 3).

Except pulp-to-fruit and peel-to-fruit weight ratio, one-way MANOVA test revealed a significant difference in all the studied parameters (Wilks' lambda = 0.00003, *p*=0.002). The harvested fruits exhibited weights ranging from 16.47 to 82.78 g, with *O. robusta* and the local ecotype displaying the highest values, while *O. dillenii* displayed the lowest weight. In terms of fruit length, the local ecotype was identified as having the longest fruit, while the AN and AA species, which belong to the same group, showed no significant difference. *Opuntia robusta* displayed the largest fruit followed by the local ecotype, while the smallest fruit was observed in the *Opuntia dillenii*. Highly significant difference was registered between the examined species. Regarding seed weight, a significant difference (*F* = 23.45, *p* < 0.01) was observed. Both *O. dillenii* and *O. robusta* displayed higher values with no significant difference between them (1.60 and 1.61, respectively), while the local ecotype recorded the lowest value. With regard to fruit size, significant differences were recorded among the studied species. *O. dillenii* is characterized by the smallest volume, while the local ecotype and *O. robusta* displayed the largest sizes. As for the pulp content, is regarded as vital factor in evaluating fruit quality. *O. robusta* and the local ecotype exhibited the highest pulp content, with no significant difference between them, while *O. dillenii* recorded the lowest pulp content. Regarding peel weight and the depth of the receptacle scar, highly significant difference was observed only between *O. dillenii* and *O. robusta*, while no significant differences were recorded between the local ecotype with each of *O. dillenii* and *O. robusta*. Despite significant differences in peel and pulp weight among the studied species, no significant difference was found in the weight ratio of peel to pulp. For the depression of the receptacle scar, the local ecotype is distinguished by the highest depression, followed by *O. robusta*, whereas *O. dillenii* registers the lowest value of 0.26 cm. A highly significant difference was observed between the local ecotype and both *O. dillenii* and *O. robusta,* whereas no significant difference was observed between *O. robusta* with *O. dillenii* (Table 3)

Upon analysing the cladodes, the statistical analysis revealed significant (*F* = 454.08, *p* < 0.05) and highly significant differences between the studied species. *O. robusta* exhibited the highest values for various cladode parameters (Table 4), including weight, width, largest distance between areoles, and thickness at tip and base, as well as the length of the longest spine. On the other hand, the local ecotype was identified as having the longest cladodes, the highest number of areoles, and the highest number of spines in the center. Meanwhile, *O. dillenii* consistently displayed the lowest values for each parameter examined, except for the length-to-width ratio, where *O. dillenii* showed the highest value, but without a significant difference compared to the local ecotype (Table 4).

To thoroughly evaluate the cultivars based on their overall profile, separate principal component analysis (PCA) of both cladodes and fruits was carried out, considering 25 quantitative traits.

**Figure 4:**
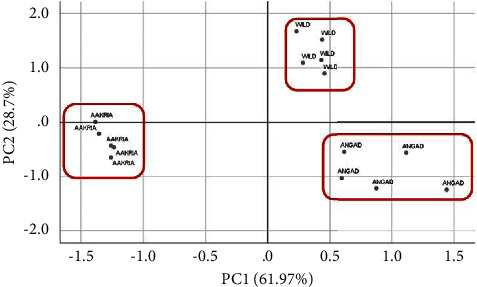
Principal component analysis of the local ecotype, *Opuntia dillenii,* and *Opuntia robusta* based on cladode quantitative descriptors. The first two components explained 61.97% and 28.7% of the variances.

The PCA of the cladodes revealed that the first two principal components explained 61.97% and 28.7% of the observed variation, respectively, accounting for a total of 98.67% of the total variation (Table supplemental 3). As depicted in Figure 4, the principal component analysis clearly demonstrated the presence of three groups.

The combination test of K-means and analysis of variance indicated that there is a significant difference between all the studied variables (Supplemental Tables 4 and 5). Cluster 2, represented by the local ecotype, was characterized by having the longest cladode, the highest number of areoles per cladode, and the highest number of areoles and spines in the center row. On the other hand, cluster 3, represented by *O. robusta*, displayed the largest cladode, the greatest thickness of the cladode at the base and tip, the largest distance between areoles, and the longest spine length. Conversely, *O. dillenii* was found to be distinct only in terms of having the highest ratio of length to width, represented in cluster 1.

**Figure 5:**
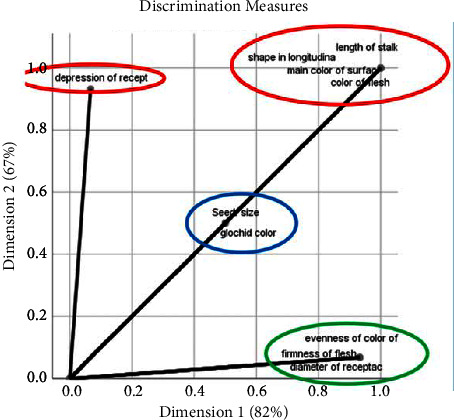
MCA (multiple correspondence analysis) dimensions discrimination measures, basing on fruit qualitative parameters conducted on the studied accessions: local prickly pear, *Opuntia robusta*, and *Opuntia dillenii*.

**Figure 6:**
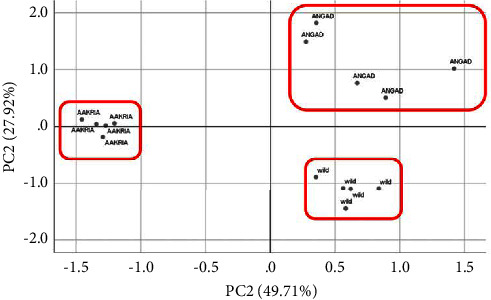
Principal component analysis of fruit quantitative variables conducted on the local ecotype, *Opuntia dillenii,* and *Opuntia robusta*.

In reference to fruits, our analysis utilizing principal component analysis (PCA) has revealed that the first two principal components account for 49.71% and 27.92% of the total variation observed, respectively (Supplemental Tables 6A–6B). Collectively, these components explain 77.64% of the total variation, thus indicating the existence of three distinct groups as depicted in Figure 5. Upon conducting K-means tests and ANOVA (Supplemental Tables 7 and 8), it has been established that despite belonging to different clusters, certain variables including peel thickness in the center, the ratio of peel weight to fruit weight, and the ratio of pulp weight to fruit weight do not demonstrate any significant difference. However, it is noteworthy that the variables that are most distinct are the largest fruit size, deepest receptacle scar, highest receptacle diameter, highest fruit and peel weight, highest pulp weight, highest weight of seeds, and largest number of fully developed seeds. The third cluster, represented by the local ecotype, is characterized by the longest fruit, the highest ratio of length to width, the highest number of areoles, the greatest value of depression of receptacle scar, and the thickest pericarp at the center (Figure 6).

### 4.2. Qualitative Morphological Traits Variability

The AFCM method was utilized to perform a qualitative data analysis on 26 traits. Cronbach's alpha index value of 0.98 surpassed the required minimum value of 0.7. Notably, the qualitative parameter analyses for fruits and cladodes were conducted separately. Specifically, for fruits, (Discrimination Measures, Figure 5) indicated that the length of the stalk, shape in the longitudinal section, primary surface color, flesh color, and depression of the receptacle scar were the most noteworthy and differentiating variables. Hence, these morphological characteristics represent the greatest variations among the studied species. In contrast, the seed size and glochid color are moderately differentiating variables, while the evenness of the surface color, flesh firmness, and receptacle scar diameter have the least impact on the morphological description.

**Figure 7:**
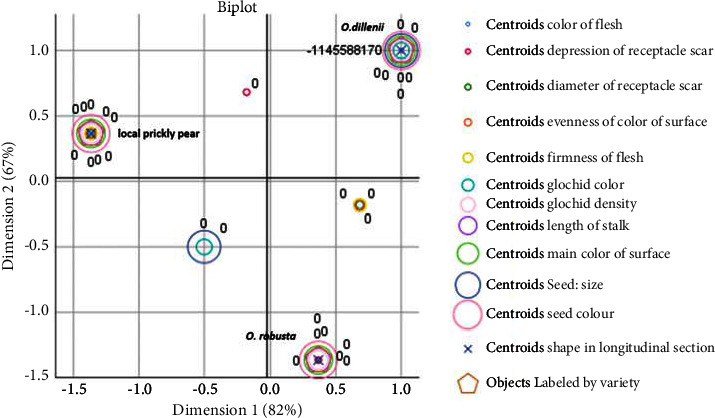
Joint plot of category points of the local prickly pear, *Opuntia robusta,* and *Opuntia dillenii*, basing on fruit qualitative parameters.

The validity of the findings was supported by the joint plot of category points (Figure 7), where the three studied species were distinguishable by flesh color, stalk length, shape in longitudinal section, primary surface color, glochid density, and seed color. Conversely, *O. robusta* demonstrated uniqueness in seed size and glochid color, while the depression of the receptacle scar was a distinguishing feature of *O. dillenii*. The local ecotype was differentiated from other species based on the diameter of the receptacle scar, flesh firmness, and evenness of the surface color.

**Figure 8:**
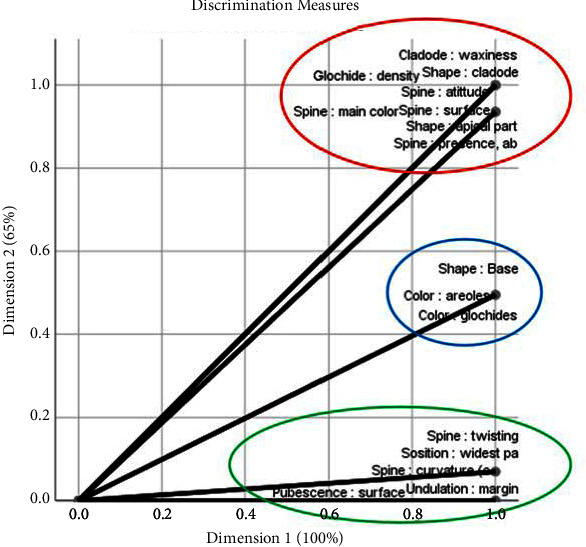
MCA (multiple correspondence analysis) dimensions discrimination measures, basing on cladode qualitative parameters conducted on the studied accessions: local prickly pear, *Opuntia robusta*, and *Opuntia dillenii*.

**Figure 9:**
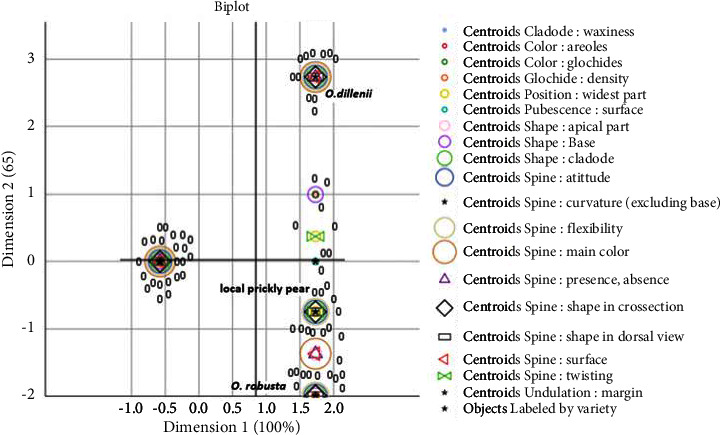
Joint plot of category points of the local prickly pear, *Opuntia robusta*, and *Opuntia dillenii*, basing on cladodes qualitative parameters.

Regarding the cladodes, the waxiness, cladode shape, apical part shape, glochid density, spine attitude, the main color of the spine, surface spine, and the presence or absence of a spine constitute the most significant morphological differences between the three species. Consequently, these reported variables are instrumental in differentiating the three species. Areole and glochid color, as well as the shape at the base, are moderately discriminating characteristics. Conversely, spine twisting, widest part position, surface pubescence, spine curvature, and undulation margin have the least impact on the morphological description (Figure 8). *O. robusta* is unique in terms of glochid color, areole color, shape at the base, and base form. Meanwhile, apical form, spine presence or absence, spine surface, and spine main color are distinct characteristics of *O. dillenii*. The local ecotype differs from the other species only in spine twisting and the position of the widest part, as illustrated in Figure 9.

## 5. Discussion

The multivariate analysis of both qualitative and quantitative parameters associated with the cladodes and fruits conclusively demonstrates a clear separation of the three studied species. Among the numerous morphological parameters examined, 13 qualitative and 23 quantitative traits were found to be significant in distinguishing the studied species.

In terms of fruits, *O. robusta* stands out due to its significant factors that greatly influence fruit quality such as higher weight, largest size, and greater pulp content, as reported in [16, 17]. Concerning the fruit size, our findings are consistent with those reported by [18] emphasizing the importance of fruit size in distinguishing and categorizing prickly pear species. These significant differences were attributed to several factors including environmental conditions [19, 20], but in this investigation, the samples of the different studied species were taken from the same location. Therefore, the observed differences cannot be linked to the environmental conditions. In this examination, the mother plants of sampled cladodes did not receive any crop management practices, such as irrigation or thinning, which could influence fruit size [21, 22]. Consequently, the observed differences are likely related to genetic factors. Our results align with [23] which discovered that fruit size is primarily determined by genetic factors, rather than environmental or edaphic factors. Furthermore, the authors of [24] asserted that Fruit size results from factors such as cell number, cell volume, and cell density, all of which are influenced by genetic factors. Besides disease resistance and productivity, fruit quality is a critical factor for consumer preference, contributing significantly to commercial success [25]. Therefore, considering fruit volume, *O. robusta* could be regarded as offering the best fruit quality as an alternative to the local prickly pear specie.

Concerning the edible portion, the pulp content serves as a key determinant of fruit quality, the greater the edible portion, the higher the fruit quality. Therefore, the content of the pulp is considered crucial in the domestication process [3]. Fruits with large sizes and a high proportion of edible pulp are highly valued, particularly for fresh consumption. According to the obtained results, *O. robusta* displayed the higher pulp weight followed by the local prickly pear, without any significant difference between them. As a result, this desirable trait makes *O. robusta* a promising substitute for the endangered ecotypes [26]. Water availability, whether through irrigation or rainwater, plays a very important role in the development of the edible part of prickly pear fruit. Notably, the pulp content is composed essentially of water (84–90%) [27]. However, as the sampling was conducted in the same geographical area, the impact of water has been excluded. Consequently, the observed difference in pulp content is likely attributed mainly to genetic variability. This aligns with the conclusion of [28], which concluded that fruit mass is genetically controlled.

Concerning seed weight, our findings are in agreement with those reported by Barbera et al. [27] who stated that prickly pear fruit quality is affected by seed content which is controlled by genotype. According to the obtained results, *O. robusta* accompanied with *O. dillenii* have the highest number of fully developed seeds, which [27] pointed out, is a significant factor in determining fruit weight. Depending on the intended use of the fruit, seed content can have a positive or negative effect. For fresh consumption, high seed content may be less desirable, while very seedy pulp is preferred for cosmetic purposes. Thus, *O. robusta* and o. dillenii may have potential applications in the cosmetics industry especially oil sector.

According to the aforementioned findings, the local ecotype possesses the thickest pericarp, making it the most resistant to physical damage, followed by *O. robusta*. Conversely, *O. dillenii* has the thinnest pericarp and may be more prone to physical damage. For nonclimacteric fruits, such as prickly pear, the suitability of the fruits for storage primarily depends on conservation conditions such as temperature and relative humidity. However, physical damage can also result in microbiological degradation during storage, thereby affecting the fruit's conservation period and quality. Therefore, two important parameters that influence fruit quality are stalk length and pericarp thickness [29]. The presence of a long peduncle facilitates fruit harvesting and prevents damage at the peduncle, which is one of the main causes of quality loss [30]. Only *O. robusta* has a short stalk, making it susceptible to physical damage. These parameters are useful in differentiating between various species of prickly pears and understanding how to effectively store and handle prickly pear fruits to maintain their quality.

Regarding receptacle scar, either for diameter or depth, no significant difference was observed. Consequently, measures related to receptacle scar do not enable the distinction between the different species of prickly pear. Our results are not in agreement with those reported by [31] who noticed that floral receptacle diameter and floral receptacle depth varied significantly among the different varieties.

All scrutinized fruit morphological characteristics have presented a promising opportunity for the potential substitution of the indigenous ecotype with the two recently introduced species. Among these, *O. Robusta* stands out as the most favorable candidate, except for its comparatively shorter stem, rendering it the primary contender for supplanting the local ecotype based on fruit-related attributes. Nevertheless, it is imperative to note that this does not imply unfavorable attributes for *O. dillenii*. On the contrary, this study underscores that *O. dillenii* possesses a distinctive feature, notably its high seed content, which holds considerable value, particularly within the cosmetic industry.

Various parameters related to cladodes allow for distinguishing between the studied species, and all the differences we found in our analyses could be attributed to genetic factors. These results are consistent with previous findings by [32]. Since the studied species and the local ecotype belong to the same region, the impact of environmental and soil factors on cladode morphology could be considered negligible. However, [33] suggested that differences in cladode size may be related to soil nutrient levels. Furthermore, observed that the presence and number of spines per areole, as well as the number of areoles, vary across different growing regions. Similarly, [34] associated the presence and the number of spines per areole with environmental conditions and gene expression.

Only *O. robusta* possesses larger-sized cladodes (length and large), whereas the cladodes of *O. dillenii* are the smallest. From a cladode point of view, in either food, fodder, or cosmetic field, *O. Robusta* emerges as the more suitable choice for replacing the local ecotype compared to *O. dillenii*.

The findings of our morphological qualitative measurements indicate conspicuous differences between the studied species. This clear separation was established by the combination of the discriminating measures and the joint plot of the category points. The former allows for the identification of the most distinctive variables, while the latter facilitates the allocation of each species to its most salient features.

Discriminant analysis showed that among the 26 phenotypic characteristics of prickly pears under examination, 13 (comprising 5 and 8 morphological descriptors of fruits and cladodes respectively) are the most discriminating. Among the fruit parameters, the length of the stalk, shape in longitudinal section, primary surface color, flesh color, and depression of the receptacle scar prove valuable in discerning between cactus pear accessions. Our results are in agreement with those reported by [35] emphasizing that surface color is one of the most discriminating parameters in distinguishing between prickly pear species. The identified morphological descriptors are easily observable with the naked eye, enhancing their effectiveness in serving as discriminant parameters.

Regarding the cladodes, the waxiness, cladode shape, apical part shape, glochid density, spine attitude, main color of spine, surface spine, and the presence or absence of a spine constitute the most significant morphological differences between the three species. Consequently, these reported variables are instrumental in differentiating the three species. Our results are in agreement with those reported by Amani et al. [35], who stated that cladode shape is one of the most discriminating morphological parameters in differentiation among the prickly pear species. Areole and glochid color, as well as the shape at the base, are moderately discriminating characteristics. Conversely, spine twisting, widest part position, surface pubescence, spine curvature, and undulation margin have the least impact on morphological description, as evidenced in Figure 9. *O. robusta* is unique in terms of glochid color, areole color, shape at the base, and base form. Meanwhile, apical form, spine presence or absence, spine surface, and spine main color are distinct characteristics of *O. dillenii*. The local ecotype differs from the other species only in spine twisting and the position of the widest part, as illustrated in Figure 9.

These outcomes align with other research that also found these qualitative traits to be useful in distinguishing between different cactus pear samples [17, 36]. The morphological qualitative traits proved effective for distinguishing among the studied species, but they did not indicate the potential for replacing the local ecotype with the two newly introduced species.

## 6. Conclusion

The present study demonstrates that the recommended morphological descriptions provided by the International Union for the Protection of New Varieties of Plants (UPOV) are effective in analysing *Opuntia* species based on their external features. The 13 quantitative and 21 qualitative descriptors used in this investigation have shown promise in discerning characteristics that allow discrimination between the studied samples. Additionally, the statistical approaches employed in this study were appropriate and useful tools for segregating prickly pear species and selecting the most discriminant parameters based on morphological descriptors. This phenological investigation has the potential to replace the local ecotype, which is threatened with extinction, by selecting species with agrophenological features suitable for industrial and socioeconomic exploitation. The recently introduced resistant species (*O. robusta* and *O. dillenii*) have potential value in the cosmetic field, and *O. robusta* has suitable morphological traits that qualify it to replace the local ecotype as far as the fruits are concerned.

The current study shows that the two resistant species (*O. robusta* and *O. dillenii*) are characterized by a higher number of fully developed seeds. Consequently, future research should focus on studying the physicochemical characteristics of the oil of the local *O. robusta* and *O. dillenii* and compare them to the local ecotype. Additionally, the organoleptic quality of *O. robusta* fruits should be studied. Overall, these findings can assist in breeding and conservation efforts for different species of cactus pear, as well as in developing management strategies for their cultivation and use.

## Figures and Tables

**Table 1:** Quantitative and qualitative pomological characters of fruit and cladode based on the UPOV test guidelines for cactus pear and xoconostle (UPOV, 2004).

Plant part	Quantitative descriptor	Qualitative descriptor	*N*
Cladode	Length (cm)	Waxiness	1
	Width (cm)	Shape	2
	Length-to-width ratio	Glochid density	3
	Thickness base (cm)	Undulation of marginal part	4
	Thickness tip (cm)	Pubescence of surface	5
	Number of areoles per cladode	Spine curvature	6
	Number of areoles in the center row	Position widest part	7
	Largest distance between areoles (cm)	Spine twisting	8
	Number of spines per areole in center	Color of glochid	9
	Number of spines in board	Color of areole	10
	Length of the longest spine (cm)	Shape base part	11
	Weight of cladode (g)	Presence of spine	12
		Shape apical part	13
		Spain surface	14
		Spain main color	15
		Spine attitude	16

Fruit	Length (cm)	Length of stalk	17
	Width (cm)	Shape in longitudinal section	18
	Ratio weight of peel per fruit	Main color of surface	19
	Ratio weight of pulp per fruit	Color of flesh	20
	Peel thickness center (cm)	Diameter of receptacle scar	21
	Seeds weight per fruit	Firmness of flesh	22
	Fruit weight (g)	Evenness of color	23
	Peel weight (g)	Glochid color	24
	Pulp weight (g)	Seed size	25
	Ratio of seeds/fruit	Depression of receptacle scar	26
	Length-to-width ratio		
	Numbers of normal seeds		
	Numbers of abortive seeds		

**Table 2:** Cladode color parameters of the local ecotype, *Opuntia dillenii*, and *Opuntia robusta*.

	*Opuntia dillenii*	*Opuntia robusta*	Local ecotype	Df	SE	*F*
L^*∗*^ (lightness)	10.33^c^	46.64^a^	20.07^b^	2	1.01	342.46^*∗∗∗*^
C (chroma saturation)	10.78^b^	12.67^b^	23.58^a^	2	1.56	19.62^*∗∗*^
H (hue)	157.85^a^	124.4^b^	117^b^	2	8.32	6.82^*∗*^

According to the analysis of variance, means (± standard error) followed by the same letters are not significantly different (*p* ≤ 0.05). ^*∗*^*p* < 0.05, ^*∗∗*^*p* < 0.01, ^*∗∗∗*^*p* < 0.001, and NS denotes not significant.

**Table 3:** Average values of one-way MANOVA of quantitative pomological traits conducted on the fruit of the *Opuntia dillenii,* local ecotype, and *Opuntia robusta*.

Fruit	*Opuntia dillenii*	Local ecotype	*Opuntia robusta*	Mean ± SE	Df	*F*
Length (cm)	4.44^b^	6.31^a^	4.62^b^	5.12 ± 0.27	2	14.6^*∗∗∗*^
Width (cm)	2.6^c^	4.29^b^	5.77^a^	4.24 ± 0.15	2	110.64^*∗∗∗*^
Ratio weight of peel/fruit	46 NS	45.93 NS	50.25 NS	48.47 ± 4.1	2	0.73^*∗*^
Ratio weight of pulp/fruit	52.82 NS	53.45 NS	49.89 NS	51.29 ± 4.2	2	0.59 NS
Peel thickness (cm)	0.27 NS	0.24 NS	0.3 NS	0.29 ± 0.4	2	2.1 NS
Seeds weight per fruit (g)	1.6^a^	0.87^b^	1.6^a^	1.36 ± 0.87	2	23.45^*∗∗∗*^
Fruit weight(g)	16.47^b^	56.25^a^	82.78^a^	51.83 ± 6.4	2	26.69^*∗∗∗*^
Peel weight (g)	7.69^c^	25.84^b^	41.6^a^	25.04 ± 3.02	2	31.39^*∗∗∗*^
Pulp weight (g)	8.7^b^	30.07^ab^	41.3^a^	26.7 ± 5.58	2	8.7^*∗∗*^
Length-to-weight ratio	1.67^b^	5.3^a^	5.2^a^	4.06 ± 0.19	2	113.35^*∗∗∗*^
Receptacle diameter scar in depth (cm)	0.45^c^	0.71^b^	1.1^a^	0.75 ± 0.62	2	27.31^*∗∗∗*^
Depression of receptacle scar (cm)	0.26^b^	0.86^a^	0.47^b^	0.53 ± 0.78	2	15.57^*∗∗∗*^

According to the analysis of variance, means (± standard error) followed by the same letters are not significantly different (*p* ≤ 0.05). ^*∗*^*p* < 0.05, ^*∗∗*^*p* < 0.01, ^*∗∗∗*^*p* < 0.001, and NS denotes not significant. (-) No significant difference was found.

**Table 4:** Average values of one-way MANOVA of the quantitative pomological characteristics conducted on the cladodes of the local ecotype, *Opuntia dillenii,* and *Opuntia robusta*.

Cladode	*Opuntia dillenii*	Local ecotype	*Opuntia robusta*	Mean ± SE	Df	*F*
Length (cm)	13.5^b^	21.52^a^	16.43^b^	17.15 ± 1.03	2	16.25^*∗∗∗*^
Width (cm)	5.35^c^	9.13^b^	15.63^a^	10.04 ± 1.17	2	87.62^*∗∗∗*^
Length-to-width ratio	2.54^a^	2.37^a^	1.05^b^	1.99 ± 0.19	2	25.25^*∗∗∗*^
Thickness base (cm)	1.03^c^	1.98^b^	2.55^a^	1.86 ± 0.19	2	17.58^*∗∗∗*^
Thickness tip (cm)	0.44^b^	0.52^b^	1.53^a^	0.83 ± 0.14	2	27.27^*∗∗∗*^
Number of areoles per cladode	38.2^c^	152.2^a^	100.2^b^	96.86 ± 12.78	2	110.64^*∗∗∗*^
Number of areoles in the center row	3^b^	6^a^	5.4^a^	4.8 ± 0.36	2	47.25^*∗∗∗*^
Largest distance between areoles (cm)	2.42^b^	1.69^c^	3.04^a^	2.38 ± 0.17	2	15.59^*∗∗∗*^
Number of spines per areole in the center row	00^c^	2.2^a^	1.6^b^	1.26 ± 0.26	2	38.8^*∗∗∗*^
Number of spines per areole in board	00^b^	3^a^	2.8^a^	1.93 ± 0.38	2	60.28^*∗∗∗*^
Length of the longest spine (cm)	00^c^	2.95^b^	3.4^a^	2.13 ± 0.41	2	292.47^*∗∗∗*^
Weight of cladode (g)	18.28^c^	70.94^b^	228.42^a^	105.88 ± 24.11	2	274.47^*∗∗∗*^

According to the analysis of variance, (± standard error) means followed by the same letters are not significantly different (*p* ≤ 0.05). ^*∗*^*p* < 0.05, ^*∗∗*^*p* < 0.01, ^*∗∗∗*^*p* < 0.001, and NS denotes not significant.

## Data Availability

The data used to support the findings of this study are included within the article.

## Supplementary Materials

Supplementary Materials The supplementary material provides additional context and detailed information supporting the findings presented in the original manuscript. This includes tables and figures that complement and enhance the understanding of the research. The supplementary material provides detailed statistical analyses. A set of supplementary tables accompanies this section, offering a comprehensive overview of statistical parameters and ensuring a thorough examination of the study's outcomes.
