# Supplementary material for: A Comparative Analysis of Morphological Characteristics between Endangered Local Prickly Pear and the Newly Introduced Dactylopius opuntiae-Resistant Species in Eastern Morocco
Source: Scientifica (Cairo). 2024 Feb 9;2024:7939465. doi: 10.1155/2024/7939465 (PMC10872770; doi:10.1155/2024/7939465)
Supplement: Supplementary Materials — The supplementary material provides additional context and detailed information supporting the findings presented in the original manuscript. This includes tables and figures that complement and enhance the understanding of the research. The supplementary material provides detailed statistical analyses. A set of supplementary tables accompanies this section, offering a comprehensive overview of statistical parameters and ensuring a thorough examination of the study's outcomes. [file 7939465.f1.zip › supplemental (1) (1).docx]

Table Supplemental 1: Climatic conditions, including average weather, temperature and precipitation of the Oujda region during the two consecutive years of study (2020-2021).

| Year | Month | Tmax (degC) | Tmin (degC) | Ppt (mm) |
| --- | --- | --- | --- | --- |
| 2020 | 1 | 14.2 | 3.1 | 25.5 |
| 2020 | 2 | 18.5 | 6.9 | 0.4 |
| 2020 | 3 | 19.9 | 8.4 | 37.7 |
| 2020 | 4 | 22.7 | 9.1 | 64 |
| 2020 | 5 | 26.4 | 13.6 | 13.3 |
| 2020 | 6 | 29.7 | 16.4 | 2.5 |
| 2020 | 7 | 34.9 | 19.4 | 2.7 |
| 2020 | 8 | 34.2 | 19.5 | 4.2 |
| 2020 | 9 | 30.4 | 15.8 | 4.8 |
| 2020 | 10 | 24.4 | 11.4 | 7 |
| 2020 | 11 | 20.5 | 10 | 12.5 |
| 2020 | 12 | 16.8 | 6.5 | 50.7 |
| 2021 | 1 | 16 | 4.9 | 19.9 |
| 2021 | 2 | 19.5 | 7.9 | 3.2 |
| 2021 | 3 | 19 | 7.4 | 44.4 |
| 2021 | 4 | 23.1 | 9.5 | 37.1 |
| 2021 | 5 | 26.3 | 13.5 | 10.7 |
| 2021 | 6 | 29.1 | 15.8 | 6 |
| 2021 | 7 | 34.7 | 19.2 | 2.5 |
| 2021 | 8 | 33.6 | 18.9 | 2.2 |
| 2021 | 9 | 30.7 | 16.1 | 19.2 |
| 2021 | 10 | 25.1 | 12.1 | 1.8 |
| 2021 | 11 | 17.2 | 6.8 | 28.4 |
| 2021 | 12 | 16.6 | 6.3 | 19.4 |

Table Supplemental 2: Cladode color parameters of local ecotype, *Opuntia dillenii* and *Opuntia robusta*.

|  | *Opuntia dillenii* | *Opuntia robusta* | Local ecotype |
| --- | --- | --- | --- |
| L*  (lightness) | 9.82 | 47.86 | 17.9 |
|  | 9.93 | 46.81 | 17.01 |
|  | 11.19 | 42.87 | 23.34 |
|  | 13.06 | 48.18 | 21.62 |
|  | 7.66 | 47.51 | 20.51 |
| C (chroma saturation) | 7.757440156 | 12.14 | 16.87 |
|  | 10.42358437 | 12.62 | 19.51 |
|  | 15.0375578 | 13.22 | 29.42 |
|  | 12.24914736 | 13.78 | 25.88 |
|  | 8.475321692 | 11.61 | 26.22 |
| H (hue) | 115.5365 | 121.05 | 116.97 |
|  | 162.239 | 127.47 | 117.96 |
|  | 155.9624 | 123.43 | 115.68 |
|  | 150.2613 | 122.44 | 117.27 |
|  | 205.2825 | 127.83 | 117.14 |
| DE | 12.26 | 30.6 | 0 |
|  | 45.75 | 32.03 | 0 |
|  | 45.44 | 26.53 | 0 |
|  | 35.76 | 29.64 | 0 |
|  | 90.82 | 32.51 | 0 |

Table Supplemental 3: Principal component analysis of local ecotype, *Opuntia dillenii* and *Opuntia robusta* based on cladode quantitative descriptors.

| **Total Variance Explained** | | | | | | | | | |
| --- | --- | --- | --- | --- | --- | --- | --- | --- | --- |
| Component | Initial Eigenvalues | | | Extraction Sums of Squared Loadings | | | Rotation Sums of Squared Loadings | | |
|  | Total | % of Variance | Cumulative % | Total | % of Variance | Cumulative % | Total | % of Variance | Cumulative % |
| 1 | 7.437 | 61.974 | 61.974 | 7.437 | 61.974 | 61.974 | 5.688 | 47.400 | 47.400 |
| 2 | 3.444 | 28.701 | 90.675 | 3.444 | 28.701 | 90.675 | 5.193 | 43.275 | 90.675 |
| 3 | .389 | 3.245 | 93.919 |  |  |  |  |  |  |
| 4 | .305 | 2.545 | 96.464 |  |  |  |  |  |  |
| 5 | .192 | 1.599 | 98.062 |  |  |  |  |  |  |
| 6 | .133 | 1.109 | 99.171 |  |  |  |  |  |  |
| 7 | .043 | .362 | 99.533 |  |  |  |  |  |  |
| 8 | .023 | .193 | 99.726 |  |  |  |  |  |  |
| 9 | .019 | .158 | 99.885 |  |  |  |  |  |  |
| 10 | .011 | .094 | 99.979 |  |  |  |  |  |  |
| 11 | .002 | .018 | 99.997 |  |  |  |  |  |  |
| 12 | .000 | .003 | 100.000 |  |  |  |  |  |  |
| Extraction Method: Principal Component Analysis. | | | | | | | | | |

Table. illustrate the Cronbach ‘s Alpha of the MCA test.

Tables Supplemental 4: K-Means Cluster analysis of cladodes quantitative variables conducted on the local ecotype, *Opuntia dillenii* and *Opuntia robusta*.

| **Final Cluster Centers** | | | |
| --- | --- | --- | --- |
|  | Cluster | | |
|  | 1 | 2 | 3 |
| Lenght | 13.50 | 21.53 | 16.44 |
| Width | 5.36 | 9.13 | 15.64 |
| Ratio lenght/width | 2.55 | 2.38 | 1.05 |
| Thikness basse | 1.04 | 1.99 | 2.55 |
| Thikness tip | .45 | .53 | 1.54 |
| Number of areole per cladode | 38.20 | 152.20 | 100.20 |
| Number of areoles in the central row | 3.00 | 6.00 | 5.40 |
| Largest distance between areoles | 2.42 | 1.70 | 3.05 |
| Number of spines per areole in central row | .00 | 2.20 | 1.60 |
| Number of spines per areole in bord | .00 | 3.00 | 2.80 |
| Length of the longest spine | .00 | 2.96 | 3.46 |
| Weight of cladode | 18.28 | 70.95 | 228.42 |

Table Supplemental 5: K-Means Cluster analysis combined to ANOVA test of cladodes quantitative variables conducted on the local ecotype, *Opuntia dillenii* and *Opuntia robusta*.

| **ANOVA** | | | | | | |
| --- | --- | --- | --- | --- | --- | --- |
|  | Cluster | | Error | | F | Sig. |
|  | Mean Square | df | Mean Square | df |  |  |
| Length | 82.389 | 2 | 5.069 | 12 | 16.255 | .000 |
| Width | 135.185 | 2 | 1.543 | 12 | 87.623 | .000 |
| Ratio lenght/width | 3.369 | 2 | .133 | 12 | 25.257 | .000 |
| Thikness basse | 2.933 | 2 | .167 | 12 | 17.588 | .000 |
| Thikness tip | 1.850 | 2 | .068 | 12 | 27.277 | .000 |
| Number of areole per cladode | 16286.667 | 2 | 147.200 | 12 | 110.643 | .000 |
| Number of areoles in the central row | 12.600 | 2 | .267 | 12 | 47.250 | .000 |
| Largest distance between areoles | 2.279 | 2 | .146 | 12 | 15.593 | .000 |
| Number of spines per areole in central row | 6.467 | 2 | .167 | 12 | 38.800 | .000 |
| Number of spines per areole in bord | 14.067 | 2 | .233 | 12 | 60.286 | .000 |
| Length of the longest spine | 17.485 | 2 | .060 | 12 | 292.476 | .000 |
| Weight of cladode | 59775.839 | 2 | 217.743 | 12 | 274.525 | .000 |
| The F tests should be used only for descriptive purposes because the clusters have been chosen to maximize the differences among cases in different clusters. The observed significance levels are not corrected for this and thus cannot be interpreted as tests of the hypothesis that the cluster means are equal. | | | | | | |

| **Total Variance Explained** | | | | | | | | | |
| --- | --- | --- | --- | --- | --- | --- | --- | --- | --- |
| Component | Initial Eigenvalues | | | Extraction Sums of Squared Loadings | | | Rotation Sums of Squared Loadings | | |
|  | Total | % of Variance | Cumulative % | Total | % of Variance | Cumulative % | Total | % of Variance | Cumulative % |
| 1 | 8.452 | 49.715 | 49.715 | 8.452 | 49.715 | 49.715 | 7.858 | 46.221 | 46.221 |
| 2 | 4.747 | 27.926 | 77.641 | 4.747 | 27.926 | 77.641 | 5.341 | 31.420 | 77.641 |
| 3 | 2.556 | 15.036 | 92.677 |  |  |  |  |  |  |
| 4 | .445 | 2.620 | 95.297 |  |  |  |  |  |  |
| 5 | .328 | 1.930 | 97.227 |  |  |  |  |  |  |
| 6 | .151 | .886 | 98.112 |  |  |  |  |  |  |
| 7 | .132 | .774 | 98.887 |  |  |  |  |  |  |
| 8 | .094 | .556 | 99.442 |  |  |  |  |  |  |
| 9 | .054 | .320 | 99.763 |  |  |  |  |  |  |
| 10 | .025 | .149 | 99.912 |  |  |  |  |  |  |
| 11 | .008 | .049 | 99.961 |  |  |  |  |  |  |
| 12 | .004 | .024 | 99.985 |  |  |  |  |  |  |
| 13 | .002 | .012 | 99.998 |  |  |  |  |  |  |
| 14 | .000 | .002 | 100.000 |  |  |  |  |  |  |
| 15 | 3.397E-16 | 1.998E-15 | 100.000 |  |  |  |  |  |  |
| 16 | -2.663E-16 | -1.566E-15 | 100.000 |  |  |  |  |  |  |
| 17 | -4.298E-16 | -2.528E-15 | 100.000 |  |  |  |  |  |  |
| Extraction Method: Principal Component Analysis. | | | | | | | | | |

Table Supplemental 6 - A: Principal component analysis of fruit quantitative variables conducted on the local ecotype, *Opuntia dillenii* and *Opuntia robusta*.

Table Supplemental 6 - B: Principal component analysis of fruit quantitative variables conducted on the local ecotype, *Opuntia dillenii* and *Opuntia robusta*.

| **Component Matrix^a^** | | |
| --- | --- | --- |
|  | Component | |
|  | 1 | 2 |
| Length | ,557 | -,782 |
| Width | ,899 | ,415 |
| Ratio length/width | ,993 | -,074 |
| Number of areoles | ,729 | -,594 |
| Depression of receptacle scar (cm) | ,669 | -,557 |
| Receptacle diameter (cm) depth | ,808 | ,457 |
| Receptacle diameter (cm) | ,835 | ,482 |
| Peel thickness center(cm) | ,060 | ,648 |
| Peel thickness base (cm) | ,815 | -,409 |
| Fruit weight (g) | ,922 | ,261 |
| Peel weight (g) | ,865 | ,434 |
| Pulp weight (g) | ,840 | ,082 |
| Ratio weight peep/fruit | ,021 | ,545 |
| Ratio weight pulp/fruit | -,018 | -,518 |
| Weight of seeds/fruit | -,362 | ,745 |
| number of fully developed seeds | ,846 | ,466 |
| number of abortive seeds | ,448 | -,815 |
| Extraction Method: Principal Component Analysis. | | |
| a. 2 components extracted. | | |

Table Supplemental 7: K-Means Cluster analysis of fruits quantitative variables conducted on the local ecotype, *Opuntia dillenii* and *Opuntia robusta*.

| **Final Cluster Centers** | | | |
| --- | --- | --- | --- |
|  | Cluster | | |
|  | 1 | 2 | 3 |
| Length | 4.62 | 4.45 | 6.31 |
| Width | 5.78 | 2.65 | 4.29 |
| Ratio length/width | 5.20 | 1.68 | 5.30 |
| Number of areoles | 30.00 | 7.40 | 68.40 |
| Depression of receptacle scar (cm) | .47 | .26 | .87 |
| Receptacle diameter (cm) depth | 1.10 | .45 | .71 |
| Receptacle diameter (cm) | 2.65 | 1.04 | 1.77 |
| Peel thickness center(cm) | .36 | .28 | .25 |
| Peel thickness base (cm) | .48 | .15 | .75 |
| Fruit weight (g) | 82.79 | 16.48 | 56.25 |
| Peel weight (g) | 41.60 | 7.70 | 25.85 |
| Pulp weight (g) | 41.30 | 8.74 | 30.08 |
| Ratio weight peep/fruit | 52.58 | 46.82 | 46.03 |
| Ratio weight pulp/fruit | 47.54 | 52.95 | 53.38 |
| Weight of seeds/fruit | 1.61 | 1.60 | .87 |
| number of fully developed seeds | 214.00 | 92.40 | 150.60 |
| number of abortive seeds | 4.00 | .20 | 194.00 |

Table Supplemental 8: K-Means Cluster analysis combined to ANOVA test of fruits quantitative variables conducted on the local ecotype, *Opuntia robusta* and *Opuntia dillenii*.

| **ANOVA** | | | | | | |
| --- | --- | --- | --- | --- | --- | --- |
|  | Cluster | | Error | | F | Sig. |
|  | Mean Square | df | Mean Square | Df |  |  |
| Length | 5.297 | 2 | .363 | 12 | 14.608 | .001 |
| Width | 12.237 | 2 | .111 | 12 | 110.465 | .000 |
| Ratio length/width | 21.281 | 2 | .188 | 12 | 113.351 | .000 |
| Number of areoles | 4755.267 | 2 | 11.700 | 12 | 406.433 | .000 |
| Depression of receptacle scar (cm) | .473 | 2 | .030 | 12 | 15.572 | .000 |
| Receptacle diameter (cm) depth | .532 | 2 | .019 | 12 | 27.318 | .000 |
| Receptacle diameter (cm) | 3.261 | 2 | .021 | 12 | 154.921 | .000 |
| Peel thickness center(cm) | .017 | 2 | .008 | 12 | 2.108 | .164 |
| Peel thickness base (cm) | .445 | 2 | .012 | 12 | 35.970 | .000 |
| Fruit weight (g) | 5569.333 | 2 | 208.635 | 12 | 26.694 | .000 |
| Peel weight (g) | 1439.583 | 2 | 45.849 | 12 | 31.398 | .000 |
| Pulp weight (g) | 1367.618 | 2 | 156.021 | 12 | 8.766 | .005 |
| Ratio weight peep/fruit | 63.918 | 2 | 86.530 | 12 | .739 | .498 |
| Ratio weight pulp/fruit | 52.912 | 2 | 88.528 | 12 | .598 | .566 |
| Weight of seeds/fruit | .898 | 2 | .038 | 12 | 23.459 | .000 |
| number of fully developed seeds | 18494.467 | 2 | 210.700 | 12 | 87.776 | .000 |
| number of abortive seeds | 61394.067 | 2 | 179.567 | 12 | 341.901 | .000 |
| The F tests should be used only for descriptive purposes because the clusters have been chosen to maximize the differences among cases in different clusters. The observed significance levels are not corrected for this and thus cannot be interpreted as tests of the hypothesis that the cluster means are equal. | | | | | | |
