# Supplementary material for: A Comparative Analysis of Morphological Characteristics between Endangered Local Prickly Pear and the Newly Introduced Dactylopius opuntiae-Resistant Species in Eastern Morocco
Source: Scientifica (Cairo). 2024 Feb 9;2024:7939465. doi: 10.1155/2024/7939465 (PMC10872770; doi:10.1155/2024/7939465)
Supplement: Supplementary Materials — The supplementary material provides additional context and detailed information supporting the findings presented in the original manuscript. This includes tables and figures that complement and enhance the understanding of the research. The supplementary material provides detailed statistical analyses. A set of supplementary tables accompanies this section, offering a comprehensive overview of statistical parameters and ensuring a thorough examination of the study's outcomes. [file 7939465.f1.zip › Supplemental 2.docx]

**Cladode**

| **Multivariate Tests^a^** | | | | | | |
| --- | --- | --- | --- | --- | --- | --- |
| Effect | | Value | F | Hypothesis df | Error df | Sig. |
| Intercept | Pillai's Trace | 1,000 | 2628,889^b^ | 12,000 | 1,000 | ,015 |
|  | Wilks' Lambda | ,000 | 2628,889^b^ | 12,000 | 1,000 | ,015 |
|  | Hotelling's Trace | 31546,673 | 2628,889^b^ | 12,000 | 1,000 | ,015 |
|  | Roy's Largest Root | 31546,673 | 2628,889^b^ | 12,000 | 1,000 | ,015 |
| variety | Pillai's Trace | 1,999 | 346,487 | 24,000 | 4,000 | ,000 |
|  | Wilks' Lambda | ,000 | 454,087^b^ | 24,000 | 2,000 | ,002 |
|  | Hotelling's Trace | 28559,607 | ,000 | 24,000 | ,000 | . |
|  | Roy's Largest Root | 27479,743 | 4579,957^c^ | 12,000 | 2,000 | ,000 |
| a. Design: Intercept + variety | | | | | | |
| b. Exact statistic | | | | | | |
| c. The statistic is an upper bound on F that yields a lower bound on the significance level. | | | | | | |

| **Tests of Between-Subjects Effects** | | | | | | |
| --- | --- | --- | --- | --- | --- | --- |
| Source | Dependent Variable | Type III Sum of Squares | df | Mean Square | F | Sig. |
| Corrected Model | Lenght | 164,778^a^ | 2 | 82,389 | 16,255 | ,000 |
|  | Width | 270,371^b^ | 2 | 135,185 | 87,623 | ,000 |
|  | Ratio lenght/width | 6,738^c^ | 2 | 3,369 | 25,257 | ,000 |
|  | Thikness basse | 5,867^d^ | 2 | 2,933 | 17,588 | ,000 |
|  | Thikness tip | 3,700^e^ | 2 | 1,850 | 27,277 | ,000 |
|  | Number of areole per cladode | 32573,333^f^ | 2 | 16286,667 | 110,643 | ,000 |
|  | Number of areoles in the central row | 25,200^g^ | 2 | 12,600 | 47,250 | ,000 |
|  | Largest distance between areoles | 4,557^h^ | 2 | 2,279 | 15,593 | ,000 |
|  | Number of spines per areole in central row | 12,933^i^ | 2 | 6,467 | 38,800 | ,000 |
|  | Number of spines per areole in bord | 28,133^j^ | 2 | 14,067 | 60,286 | ,000 |
|  | Length of the longest spine | 34,969^k^ | 2 | 17,485 | 292,476 | ,000 |
|  | Weight of cladode | 119551,678^l^ | 2 | 59775,839 | 274,525 | ,000 |
| Intercept | Lenght | 4414,959 | 1 | 4414,959 | 871,029 | ,000 |
|  | Width | 1513,088 | 1 | 1513,088 | 980,742 | ,000 |
|  | Ratio lenght/width | 59,561 | 1 | 59,561 | 446,495 | ,000 |
|  | Thikness basse | 51,916 | 1 | 51,916 | 311,261 | ,000 |
|  | Thikness tip | 10,497 | 1 | 10,497 | 154,780 | ,000 |
|  | Number of areole per cladode | 140747,267 | 1 | 140747,267 | 956,163 | ,000 |
|  | Number of areoles in the central row | 345,600 | 1 | 345,600 | 1296,000 | ,000 |
|  | Largest distance between areoles | 85,543 | 1 | 85,543 | 585,382 | ,000 |
|  | Number of spines per areole in central row | 24,067 | 1 | 24,067 | 144,400 | ,000 |
|  | Number of spines per areole in bord | 56,067 | 1 | 56,067 | 240,286 | ,000 |
|  | Length of the longest spine | 68,677 | 1 | 68,677 | 1148,795 | ,000 |
|  | Weight of cladode | 168169,204 | 1 | 168169,204 | 772,330 | ,000 |
| variety | Lenght | 164,778 | 2 | 82,389 | 16,255 | ,000 |
|  | Width | 270,371 | 2 | 135,185 | 87,623 | ,000 |
|  | Ratio lenght/width | 6,738 | 2 | 3,369 | 25,257 | ,000 |
|  | Thikness basse | 5,867 | 2 | 2,933 | 17,588 | ,000 |
|  | Thikness tip | 3,700 | 2 | 1,850 | 27,277 | ,000 |
|  | Number of areole per cladode | 32573,333 | 2 | 16286,667 | 110,643 | ,000 |
|  | Number of areoles in the central row | 25,200 | 2 | 12,600 | 47,250 | ,000 |
|  | Largest distance between areoles | 4,557 | 2 | 2,279 | 15,593 | ,000 |
|  | Number of spines per areole in central row | 12,933 | 2 | 6,467 | 38,800 | ,000 |
|  | Number of spines per areole in bord | 28,133 | 2 | 14,067 | 60,286 | ,000 |
|  | Length of the longest spine | 34,969 | 2 | 17,485 | 292,476 | ,000 |
|  | Weight of cladode | 119551,678 | 2 | 59775,839 | 274,525 | ,000 |
| Error | Lenght | 60,824 | 12 | 5,069 |  |  |
|  | Width | 18,514 | 12 | 1,543 |  |  |
|  | Ratio lenght/width | 1,601 | 12 | ,133 |  |  |
|  | Thikness basse | 2,002 | 12 | ,167 |  |  |
|  | Thikness tip | ,814 | 12 | ,068 |  |  |
|  | Number of areole per cladode | 1766,400 | 12 | 147,200 |  |  |
|  | Number of areoles in the central row | 3,200 | 12 | ,267 |  |  |
|  | Largest distance between areoles | 1,754 | 12 | ,146 |  |  |
|  | Number of spines per areole in central row | 2,000 | 12 | ,167 |  |  |
|  | Number of spines per areole in bord | 2,800 | 12 | ,233 |  |  |
|  | Length of the longest spine | ,717 | 12 | ,060 |  |  |
|  | Weight of cladode | 2612,912 | 12 | 217,743 |  |  |
| Total | Lenght | 4640,561 | 15 |  |  |  |
|  | Width | 1801,973 | 15 |  |  |  |
|  | Ratio lenght/width | 67,900 | 15 |  |  |  |
|  | Thikness basse | 59,785 | 15 |  |  |  |
|  | Thikness tip | 15,010 | 15 |  |  |  |
|  | Number of areole per cladode | 175087,000 | 15 |  |  |  |
|  | Number of areoles in the central row | 374,000 | 15 |  |  |  |
|  | Largest distance between areoles | 91,854 | 15 |  |  |  |
|  | Number of spines per areole in central row | 39,000 | 15 |  |  |  |
|  | Number of spines per areole in bord | 87,000 | 15 |  |  |  |
|  | Length of the longest spine | 104,364 | 15 |  |  |  |
|  | Weight of cladode | 290333,795 | 15 |  |  |  |
| Corrected Total | Lenght | 225,602 | 14 |  |  |  |
|  | Width | 288,884 | 14 |  |  |  |
|  | Ratio lenght/width | 8,339 | 14 |  |  |  |
|  | Thikness basse | 7,869 | 14 |  |  |  |
|  | Thikness tip | 4,514 | 14 |  |  |  |
|  | Number of areole per cladode | 34339,733 | 14 |  |  |  |
|  | Number of areoles in the central row | 28,400 | 14 |  |  |  |
|  | Largest distance between areoles | 6,311 | 14 |  |  |  |
|  | Number of spines per areole in central row | 14,933 | 14 |  |  |  |
|  | Number of spines per areole in bord | 30,933 | 14 |  |  |  |
|  | Length of the longest spine | 35,687 | 14 |  |  |  |
|  | Weight of cladode | 122164,590 | 14 |  |  |  |
| a. R Squared = ,730 (Adjusted R Squared = ,685) | | | | | | |
| b. R Squared = ,936 (Adjusted R Squared = ,925) | | | | | | |
| c. R Squared = ,808 (Adjusted R Squared = ,776) | | | | | | |
| d. R Squared = ,746 (Adjusted R Squared = ,703) | | | | | | |
| e. R Squared = ,820 (Adjusted R Squared = ,790) | | | | | | |
| f. R Squared = ,949 (Adjusted R Squared = ,940) | | | | | | |
| g. R Squared = ,887 (Adjusted R Squared = ,869) | | | | | | |
| h. R Squared = ,722 (Adjusted R Squared = ,676) | | | | | | |
| i. R Squared = ,866 (Adjusted R Squared = ,844) | | | | | | |
| j. R Squared = ,909 (Adjusted R Squared = ,894) | | | | | | |
| k. R Squared = ,980 (Adjusted R Squared = ,977) | | | | | | |
| l. R Squared = ,979 (Adjusted R Squared = ,975) | | | | | | |

| **Multiple Comparisons** | | | | | | | |
| --- | --- | --- | --- | --- | --- | --- | --- |
| Tamhane | | | | | | | |
| Dependent Variable | (I) Variety | (J) Variety | Mean Difference (I-J) | Std. Error | Sig. | 95% Confidence Interval | |
|  |  |  |  |  |  | Lower Bound | Upper Bound |
| Lenght | Opuntia robusta | Opuntia dillenii | 2,9310 | ,93346 | ,081 | -,4348 | 6,2968 |
|  |  | Local ecotype | -5,0912 | 1,72252 | ,067 | -10,5570 | ,3746 |
|  | Opuntia dillenii | Opuntia robusta | -2,9310 | ,93346 | ,081 | -6,2968 | ,4348 |
|  |  | Local ecotype | -8,0222^*^ | 1,49799 | ,014 | -13,7112 | -2,3332 |
|  | Local ecotype | Opuntia robusta | 5,0912 | 1,72252 | ,067 | -,3746 | 10,5570 |
|  |  | Opuntia dillenii | 8,0222^*^ | 1,49799 | ,014 | 2,3332 | 13,7112 |
| Width | Opuntia robusta | Opuntia dillenii | 10,2790^*^ | ,93670 | ,001 | 6,8411 | 13,7169 |
|  |  | Local ecotype | 6,5062^*^ | ,93135 | ,004 | 3,0513 | 9,9611 |
|  | Opuntia dillenii | Opuntia robusta | -10,2790^*^ | ,93670 | ,001 | -13,7169 | -6,8411 |
|  |  | Local ecotype | -3,7728^*^ | ,32641 | ,000 | -4,7557 | -2,7899 |
|  | Local ecotype | Opuntia robusta | -6,5062^*^ | ,93135 | ,004 | -9,9611 | -3,0513 |
|  |  | Opuntia dillenii | 3,7728^*^ | ,32641 | ,000 | 2,7899 | 4,7557 |
| Ratio lenght/width | Opuntia robusta | Opuntia dillenii | -1,4998^*^ | ,17540 | ,002 | -2,1428 | -,8568 |
|  |  | Local ecotype | -1,3282^*^ | ,22661 | ,010 | -2,1821 | -,4744 |
|  | Opuntia dillenii | Opuntia robusta | 1,4998^*^ | ,17540 | ,002 | ,8568 | 2,1428 |
|  |  | Local ecotype | ,1715 | ,27921 | ,913 | -,6823 | 1,0253 |
|  | Local ecotype | Opuntia robusta | 1,3282^*^ | ,22661 | ,010 | ,4744 | 2,1821 |
|  |  | Opuntia dillenii | -,1715 | ,27921 | ,913 | -1,0253 | ,6823 |
| Thikness basse | Opuntia robusta | Opuntia dillenii | 1,5160^*^ | ,28251 | ,015 | ,4409 | 2,5911 |
|  |  | Local ecotype | ,5672 | ,31240 | ,318 | -,4579 | 1,5923 |
|  | Opuntia dillenii | Opuntia robusta | -1,5160^*^ | ,28251 | ,015 | -2,5911 | -,4409 |
|  |  | Local ecotype | -,9488^*^ | ,15082 | ,005 | -1,4810 | -,4166 |
|  | Local ecotype | Opuntia robusta | -,5672 | ,31240 | ,318 | -1,5923 | ,4579 |
|  |  | Opuntia dillenii | ,9488^*^ | ,15082 | ,005 | ,4166 | 1,4810 |
| Thikness tip | Opuntia robusta | Opuntia dillenii | 1,0908^*^ | ,20139 | ,014 | ,3293 | 1,8523 |
|  |  | Local ecotype | 1,0118^*^ | ,19795 | ,020 | ,2348 | 1,7888 |
|  | Opuntia dillenii | Opuntia robusta | -1,0908^*^ | ,20139 | ,014 | -1,8523 | -,3293 |
|  |  | Local ecotype | -,0790 | ,04047 | ,300 | -,2255 | ,0675 |
|  | Local ecotype | Opuntia robusta | -1,0118^*^ | ,19795 | ,020 | -1,7888 | -,2348 |
|  |  | Opuntia dillenii | ,0790 | ,04047 | ,300 | -,0675 | ,2255 |
| Number of areole per cladode | Opuntia robusta | Opuntia dillenii | 62,0000^*^ | 1,78326 | ,000 | 56,4097 | 67,5903 |
|  |  | Local ecotype | -52,0000^*^ | 9,34773 | ,013 | -87,7849 | -16,2151 |
|  | Opuntia dillenii | Opuntia robusta | -62,0000^*^ | 1,78326 | ,000 | -67,5903 | -56,4097 |
|  |  | Local ecotype | -114,0000^*^ | 9,27793 | ,001 | -150,1038 | -77,8962 |
|  | Local ecotype | Opuntia robusta | 52,0000^*^ | 9,34773 | ,013 | 16,2151 | 87,7849 |
|  |  | Opuntia dillenii | 114,0000^*^ | 9,27793 | ,001 | 77,8962 | 150,1038 |
| Number of areoles in the central row | Opuntia robusta | Opuntia dillenii | 2,4000^*^ | ,40000 | ,012 | ,8238 | 3,9762 |
|  |  | Local ecotype | -,6000 | ,40000 | ,503 | -2,1762 | ,9762 |
|  | Opuntia dillenii | Opuntia robusta | -2,4000^*^ | ,40000 | ,012 | -3,9762 | -,8238 |
|  |  | Local ecotype | -3,0000 | ,00000 | . | -3,0000 | -3,0000 |
|  | Local ecotype | Opuntia robusta | ,6000 | ,40000 | ,503 | -,9762 | 2,1762 |
|  |  | Opuntia dillenii | 3,0000 | ,00000 | . | 3,0000 | 3,0000 |
| Largest distance between areoles | Opuntia robusta | Opuntia dillenii | ,6220 | ,29112 | ,216 | -,3412 | 1,5852 |
|  |  | Local ecotype | 1,3488^*^ | ,26681 | ,017 | ,3442 | 2,3534 |
|  | Opuntia dillenii | Opuntia robusta | -,6220 | ,29112 | ,216 | -1,5852 | ,3412 |
|  |  | Local ecotype | ,7268^*^ | ,13935 | ,008 | ,2513 | 1,2023 |
|  | Local ecotype | Opuntia robusta | -1,3488^*^ | ,26681 | ,017 | -2,3534 | -,3442 |
|  |  | Opuntia dillenii | -,7268^*^ | ,13935 | ,008 | -1,2023 | -,2513 |
| Number of spines per areole in central row | Opuntia robusta | Opuntia dillenii | 1,6000^*^ | ,24495 | ,008 | ,6348 | 2,5652 |
|  |  | Local ecotype | -,6000 | ,31623 | ,261 | -1,5597 | ,3597 |
|  | Opuntia dillenii | Opuntia robusta | -1,6000^*^ | ,24495 | ,008 | -2,5652 | -,6348 |
|  |  | Local ecotype | -2,2000^*^ | ,20000 | ,001 | -2,9881 | -1,4119 |
|  | Local ecotype | Opuntia robusta | ,6000 | ,31623 | ,261 | -,3597 | 1,5597 |
|  |  | Opuntia dillenii | 2,2000^*^ | ,20000 | ,001 | 1,4119 | 2,9881 |
| Number of spines per areole in bord | Opuntia robusta | Opuntia dillenii | 2,8000^*^ | ,37417 | ,005 | 1,3256 | 4,2744 |
|  |  | Local ecotype | -,2000 | ,37417 | ,946 | -1,6744 | 1,2744 |
|  | Opuntia dillenii | Opuntia robusta | -2,8000^*^ | ,37417 | ,005 | -4,2744 | -1,3256 |
|  |  | Local ecotype | -3,0000 | ,00000 | . | -3,0000 | -3,0000 |
|  | Local ecotype | Opuntia robusta | ,2000 | ,37417 | ,946 | -1,2744 | 1,6744 |
|  |  | Opuntia dillenii | 3,0000 | ,00000 | . | 3,0000 | 3,0000 |
| Length of the longest spine | Opuntia robusta | Opuntia dillenii | 3,4608^*^ | ,17608 | ,000 | 2,7670 | 4,1546 |
|  |  | Local ecotype | ,5024 | ,18939 | ,124 | -,1514 | 1,1562 |
|  | Opuntia dillenii | Opuntia robusta | -3,4608^*^ | ,17608 | ,000 | -4,1546 | -2,7670 |
|  |  | Local ecotype | -2,9584^*^ | ,06976 | ,000 | -3,2333 | -2,6835 |
|  | Local ecotype | Opuntia robusta | -,5024 | ,18939 | ,124 | -1,1562 | ,1514 |
|  |  | Opuntia dillenii | 2,9584^*^ | ,06976 | ,000 | 2,6835 | 3,2333 |
| Weight of cladode | Opuntia robusta | Opuntia dillenii | 210,1400^*^ | 8,25661 | ,000 | 179,2196 | 241,0604 |
|  |  | Local ecotype | 157,4760^*^ | 11,29095 | ,000 | 123,5478 | 191,4042 |
|  | Opuntia dillenii | Opuntia robusta | -210,1400^*^ | 8,25661 | ,000 | -241,0604 | -179,2196 |
|  |  | Local ecotype | -52,6640^*^ | 8,10149 | ,006 | -82,9476 | -22,3804 |
|  | Local ecotype | Opuntia robusta | -157,4760^*^ | 11,29095 | ,000 | -191,4042 | -123,5478 |
|  |  | Opuntia dillenii | 52,6640^*^ | 8,10149 | ,006 | 22,3804 | 82,9476 |
| Based on observed means.  The error term is Mean Square(Error) = 217,743. | | | | | | | |
| *. The mean difference is significant at the ,05 level. | | | | | | | |

**Fruit**

| **Multivariate Tests^a^** | | | | | | |
| --- | --- | --- | --- | --- | --- | --- |
| Effect | | Value | F | Hypothesis df | Error df | Sig. |
| Intercept | Pillai's Trace | 1,000 | 256,893^b^ | 12,000 | 1,000 | ,049 |
|  | Wilks' Lambda | ,000 | 256,893^b^ | 12,000 | 1,000 | ,049 |
|  | Hotelling's Trace | 3082,720 | 256,893^b^ | 12,000 | 1,000 | ,049 |
|  | Roy's Largest Root | 3082,720 | 256,893^b^ | 12,000 | 1,000 | ,049 |
| cultivar | Pillai's Trace | 1,997 | 112,098 | 24,000 | 4,000 | ,000 |
|  | Wilks' Lambda | ,000 | 59,362^b^ | 24,000 | 2,000 | ,017 |
|  | Hotelling's Trace | 1508,911 | ,000 | 24,000 | ,000 | . |
|  | Roy's Largest Root | 1003,146 | 167,191^c^ | 12,000 | 2,000 | ,006 |
| a. Design: Intercept + cultivar | | | | | | |
| b. Exact statistic | | | | | | |
| c. The statistic is an upper bound on F that yields a lower bound on the significance level. | | | | | | |

| **Tests of Between-Subjects Effects** | | | | | | |
| --- | --- | --- | --- | --- | --- | --- |
| Source | Dependent Variable | Type III Sum of Squares | df | Mean Square | F | Sig. |
| Corrected Model | Length | 10,595^a^ | 2 | 5,297 | 14,608 | ,001 |
|  | Width | 24,473^b^ | 2 | 12,237 | 110,465 | ,000 |
|  | Ratio length/width | 42,562^c^ | 2 | 21,281 | 113,351 | ,000 |
|  | Number of areoles | 9510,533^d^ | 2 | 4755,267 | 406,433 | ,000 |
|  | Depression of receptacle scar (cm) | ,946^e^ | 2 | ,473 | 15,572 | ,000 |
|  | Receptacle diameter (cm) depth | 1,065^f^ | 2 | ,532 | 27,318 | ,000 |
|  | Receptacle diameter (cm) | 6,523^g^ | 2 | 3,261 | 154,921 | ,000 |
|  | Peel thickness center(cm) | ,034^h^ | 2 | ,017 | 2,108 | ,164 |
|  | Peel thickness base (cm) | ,890^i^ | 2 | ,445 | 35,970 | ,000 |
|  | Fruit weight (g) | 11138,666^j^ | 2 | 5569,333 | 26,694 | ,000 |
|  | Peel weight (g) | 2879,165^k^ | 2 | 1439,583 | 31,398 | ,000 |
|  | Pulp weight (g) | 2735,235^l^ | 2 | 1367,618 | 8,766 | ,005 |
|  | Ratio weight peep/fruit | 127,835^m^ | 2 | 63,918 | ,739 | ,498 |
|  | Ratio weight pulp/fruit | 105,825^n^ | 2 | 52,912 | ,598 | ,566 |
|  | Weight of seeds/fruit | 1,796^o^ | 2 | ,898 | 23,459 | ,000 |
|  | number of fully developed seeds | 36988,933^p^ | 2 | 18494,467 | 87,776 | ,000 |
|  | number of abortive seeds | 122788,133^q^ | 2 | 61394,067 | 341,901 | ,000 |
| Intercept | Length | 394,446 | 1 | 394,446 | 1087,695 | ,000 |
|  | Width | 269,766 | 1 | 269,766 | 2435,286 | ,000 |
|  | Ratio length/width | 247,415 | 1 | 247,415 | 1317,842 | ,000 |
|  | Number of areoles | 18656,067 | 1 | 18656,067 | 1594,536 | ,000 |
|  | Depression of receptacle scar (cm) | 4,246 | 1 | 4,246 | 139,733 | ,000 |
|  | Receptacle diameter (cm) depth | 8,576 | 1 | 8,576 | 440,099 | ,000 |
|  | Receptacle diameter (cm) | 49,537 | 1 | 49,537 | 2353,005 | ,000 |
|  | Peel thickness center(cm) | 1,295 | 1 | 1,295 | 162,879 | ,000 |
|  | Peel thickness base (cm) | 3,195 | 1 | 3,195 | 258,301 | ,000 |
|  | Fruit weight (g) | 40307,674 | 1 | 40307,674 | 193,197 | ,000 |
|  | Peel weight (g) | 9412,037 | 1 | 9412,037 | 205,281 | ,000 |
|  | Pulp weight (g) | 10698,157 | 1 | 10698,157 | 68,569 | ,000 |
|  | Ratio weight peep/fruit | 35253,898 | 1 | 35253,898 | 407,419 | ,000 |
|  | Ratio weight pulp/fruit | 39462,846 | 1 | 39462,846 | 445,768 | ,000 |
|  | Weight of seeds/fruit | 27,798 | 1 | 27,798 | 726,187 | ,000 |
|  | number of fully developed seeds | 348081,667 | 1 | 348081,667 | 1652,025 | ,000 |
|  | number of abortive seeds | 65472,067 | 1 | 65472,067 | 364,611 | ,000 |
| cultivar | Length | 10,595 | 2 | 5,297 | 14,608 | ,001 |
|  | Width | 24,473 | 2 | 12,237 | 110,465 | ,000 |
|  | Ratio length/width | 42,562 | 2 | 21,281 | 113,351 | ,000 |
|  | Number of areoles | 9510,533 | 2 | 4755,267 | 406,433 | ,000 |
|  | Depression of receptacle scar (cm) | ,946 | 2 | ,473 | 15,572 | ,000 |
|  | Receptacle diameter (cm) depth | 1,065 | 2 | ,532 | 27,318 | ,000 |
|  | Receptacle diameter (cm) | 6,523 | 2 | 3,261 | 154,921 | ,000 |
|  | Peel thickness center(cm) | ,034 | 2 | ,017 | 2,108 | ,164 |
|  | Peel thickness base (cm) | ,890 | 2 | ,445 | 35,970 | ,000 |
|  | Fruit weight (g) | 11138,666 | 2 | 5569,333 | 26,694 | ,000 |
|  | Peel weight (g) | 2879,165 | 2 | 1439,583 | 31,398 | ,000 |
|  | Pulp weight (g) | 2735,235 | 2 | 1367,618 | 8,766 | ,005 |
|  | Ratio weight peep/fruit | 127,835 | 2 | 63,918 | ,739 | ,498 |
|  | Ratio weight pulp/fruit | 105,825 | 2 | 52,912 | ,598 | ,566 |
|  | Weight of seeds/fruit | 1,796 | 2 | ,898 | 23,459 | ,000 |
|  | number of fully developed seeds | 36988,933 | 2 | 18494,467 | 87,776 | ,000 |
|  | number of abortive seeds | 122788,133 | 2 | 61394,067 | 341,901 | ,000 |
| Error | Length | 4,352 | 12 | ,363 |  |  |
|  | Width | 1,329 | 12 | ,111 |  |  |
|  | Ratio length/width | 2,253 | 12 | ,188 |  |  |
|  | Number of areoles | 140,400 | 12 | 11,700 |  |  |
|  | Depression of receptacle scar (cm) | ,365 | 12 | ,030 |  |  |
|  | Receptacle diameter (cm) depth | ,234 | 12 | ,019 |  |  |
|  | Receptacle diameter (cm) | ,253 | 12 | ,021 |  |  |
|  | Peel thickness center(cm) | ,095 | 12 | ,008 |  |  |
|  | Peel thickness base (cm) | ,148 | 12 | ,012 |  |  |
|  | Fruit weight (g) | 2503,621 | 12 | 208,635 |  |  |
|  | Peel weight (g) | 550,194 | 12 | 45,849 |  |  |
|  | Pulp weight (g) | 1872,257 | 12 | 156,021 |  |  |
|  | Ratio weight peep/fruit | 1038,359 | 12 | 86,530 |  |  |
|  | Ratio weight pulp/fruit | 1062,333 | 12 | 88,528 |  |  |
|  | Weight of seeds/fruit | ,459 | 12 | ,038 |  |  |
|  | number of fully developed seeds | 2528,400 | 12 | 210,700 |  |  |
|  | number of abortive seeds | 2154,800 | 12 | 179,567 |  |  |
| Total | Length | 409,392 | 15 |  |  |  |
|  | Width | 295,568 | 15 |  |  |  |
|  | Ratio length/width | 292,229 | 15 |  |  |  |
|  | Number of areoles | 28307,000 | 15 |  |  |  |
|  | Depression of receptacle scar (cm) | 5,558 | 15 |  |  |  |
|  | Receptacle diameter (cm) depth | 9,875 | 15 |  |  |  |
|  | Receptacle diameter (cm) | 56,312 | 15 |  |  |  |
|  | Peel thickness center(cm) | 1,424 | 15 |  |  |  |
|  | Peel thickness base (cm) | 4,234 | 15 |  |  |  |
|  | Fruit weight (g) | 53949,960 | 15 |  |  |  |
|  | Peel weight (g) | 12841,395 | 15 |  |  |  |
|  | Pulp weight (g) | 15305,649 | 15 |  |  |  |
|  | Ratio weight peep/fruit | 36420,092 | 15 |  |  |  |
|  | Ratio weight pulp/fruit | 40631,004 | 15 |  |  |  |
|  | Weight of seeds/fruit | 30,054 | 15 |  |  |  |
|  | number of fully developed seeds | 387599,000 | 15 |  |  |  |
|  | number of abortive seeds | 190415,000 | 15 |  |  |  |
| Corrected Total | Length | 14,946 | 14 |  |  |  |
|  | Width | 25,803 | 14 |  |  |  |
|  | Ratio length/width | 44,814 | 14 |  |  |  |
|  | Number of areoles | 9650,933 | 14 |  |  |  |
|  | Depression of receptacle scar (cm) | 1,311 | 14 |  |  |  |
|  | Receptacle diameter (cm) depth | 1,299 | 14 |  |  |  |
|  | Receptacle diameter (cm) | 6,776 | 14 |  |  |  |
|  | Peel thickness center(cm) | ,129 | 14 |  |  |  |
|  | Peel thickness base (cm) | 1,038 | 14 |  |  |  |
|  | Fruit weight (g) | 13642,287 | 14 |  |  |  |
|  | Peel weight (g) | 3429,359 | 14 |  |  |  |
|  | Pulp weight (g) | 4607,492 | 14 |  |  |  |
|  | Ratio weight peep/fruit | 1166,194 | 14 |  |  |  |
|  | Ratio weight pulp/fruit | 1168,158 | 14 |  |  |  |
|  | Weight of seeds/fruit | 2,255 | 14 |  |  |  |
|  | number of fully developed seeds | 39517,333 | 14 |  |  |  |
|  | number of abortive seeds | 124942,933 | 14 |  |  |  |
| a. R Squared = ,709 (Adjusted R Squared = ,660) | | | | | | |
| b. R Squared = ,948 (Adjusted R Squared = ,940) | | | | | | |
| c. R Squared = ,950 (Adjusted R Squared = ,941) | | | | | | |
| d. R Squared = ,985 (Adjusted R Squared = ,983) | | | | | | |
| e. R Squared = ,722 (Adjusted R Squared = ,676) | | | | | | |
| f. R Squared = ,820 (Adjusted R Squared = ,790) | | | | | | |
| g. R Squared = ,963 (Adjusted R Squared = ,957) | | | | | | |
| h. R Squared = ,260 (Adjusted R Squared = ,137) | | | | | | |
| i. R Squared = ,857 (Adjusted R Squared = ,833) | | | | | | |
| j. R Squared = ,816 (Adjusted R Squared = ,786) | | | | | | |
| k. R Squared = ,840 (Adjusted R Squared = ,813) | | | | | | |
| l. R Squared = ,594 (Adjusted R Squared = ,526) | | | | | | |
| m. R Squared = ,110 (Adjusted R Squared = -,039) | | | | | | |
| n. R Squared = ,091 (Adjusted R Squared = -,061) | | | | | | |
| o. R Squared = ,796 (Adjusted R Squared = ,762) | | | | | | |
| p. R Squared = ,936 (Adjusted R Squared = ,925) | | | | | | |
| q. R Squared = ,983 (Adjusted R Squared = ,980) | | | | | | |

| **Multiple Comparisons** | | | | | | | |
| --- | --- | --- | --- | --- | --- | --- | --- |
| Tamhane | | | | | | | |
| Dependent Variable | (I) cultivar | (J) cultivar | Mean Difference (I-J) | Std. Error | Sig. | 95% Confidence Interval | |
|  |  |  |  |  |  | Lower Bound | Upper Bound |
| Length | Local ecotype | Opuntia dillenii | 1,8642^*^ | ,20906 | ,002 | 1,0788 | 2,6496 |
|  |  | Opuntia robusta | 1,6884^*^ | ,46443 | ,034 | ,1511 | 3,2257 |
|  | Opuntia dillenii | Local ecotype | -1,8642^*^ | ,20906 | ,002 | -2,6496 | -1,0788 |
|  |  | Opuntia robusta | -,1758 | ,41926 | ,972 | -1,8075 | 1,4559 |
|  | Opuntia robusta | Local ecotype | -1,6884^*^ | ,46443 | ,034 | -3,2257 | -,1511 |
|  |  | Opuntia dillenii | ,1758 | ,41926 | ,972 | -1,4559 | 1,8075 |
| Width | Local ecotype | Opuntia dillenii | 1,6444^*^ | ,17255 | ,001 | 1,0051 | 2,2837 |
|  |  | Opuntia robusta | -1,4830^*^ | ,25450 | ,001 | -2,2511 | -,7149 |
|  | Opuntia dillenii | Local ecotype | -1,6444^*^ | ,17255 | ,001 | -2,2837 | -1,0051 |
|  |  | Opuntia robusta | -3,1274^*^ | ,19592 | ,000 | -3,8628 | -2,3920 |
|  | Opuntia robusta | Local ecotype | 1,4830^*^ | ,25450 | ,001 | ,7149 | 2,2511 |
|  |  | Opuntia dillenii | 3,1274^*^ | ,19592 | ,000 | 2,3920 | 3,8628 |
| Ratio length/width | Local ecotype | Opuntia dillenii | 3,6235^*^ | ,15484 | ,000 | 3,0371 | 4,2100 |
|  |  | Opuntia robusta | ,1027 | ,33435 | ,988 | -,9950 | 1,2004 |
|  | Opuntia dillenii | Local ecotype | -3,6235^*^ | ,15484 | ,000 | -4,2100 | -3,0371 |
|  |  | Opuntia robusta | -3,5208^*^ | ,29921 | ,001 | -4,6870 | -2,3547 |
|  | Opuntia robusta | Local ecotype | -,1027 | ,33435 | ,988 | -1,2004 | ,9950 |
|  |  | Opuntia dillenii | 3,5208^*^ | ,29921 | ,001 | 2,3547 | 4,6870 |
| Number of areoles | Local ecotype | Opuntia dillenii | 61,0000^*^ | 1,97990 | ,000 | 53,9053 | 68,0947 |
|  |  | Opuntia robusta | 38,4000^*^ | 2,58070 | ,000 | 30,6368 | 46,1632 |
|  | Opuntia dillenii | Local ecotype | -61,0000^*^ | 1,97990 | ,000 | -68,0947 | -53,9053 |
|  |  | Opuntia robusta | -22,6000^*^ | 1,86011 | ,000 | -29,1933 | -16,0067 |
|  | Opuntia robusta | Local ecotype | -38,4000^*^ | 2,58070 | ,000 | -46,1632 | -30,6368 |
|  |  | Opuntia dillenii | 22,6000^*^ | 1,86011 | ,000 | 16,0067 | 29,1933 |
| Depression of receptacle scar (cm) | Local ecotype | Opuntia dillenii | ,6062^*^ | ,10215 | ,004 | ,2668 | ,9456 |
|  |  | Opuntia robusta | ,3944^*^ | ,12756 | ,044 | ,0110 | ,7778 |
|  | Opuntia dillenii | Local ecotype | -,6062^*^ | ,10215 | ,004 | -,9456 | -,2668 |
|  |  | Opuntia robusta | -,2118 | ,09880 | ,213 | -,5373 | ,1137 |
|  | Opuntia robusta | Local ecotype | -,3944^*^ | ,12756 | ,044 | -,7778 | -,0110 |
|  |  | Opuntia dillenii | ,2118 | ,09880 | ,213 | -,1137 | ,5373 |
| Receptacle diameter (cm) depth | Local ecotype | Opuntia dillenii | ,2556 | ,07511 | ,064 | -,0186 | ,5298 |
|  |  | Opuntia robusta | -,3922^*^ | ,08034 | ,018 | -,6881 | -,0963 |
|  | Opuntia dillenii | Local ecotype | -,2556 | ,07511 | ,064 | -,5298 | ,0186 |
|  |  | Opuntia robusta | -,6478^*^ | ,10624 | ,001 | -,9674 | -,3282 |
|  | Opuntia robusta | Local ecotype | ,3922^*^ | ,08034 | ,018 | ,0963 | ,6881 |
|  |  | Opuntia dillenii | ,6478^*^ | ,10624 | ,001 | ,3282 | ,9674 |
| Receptacle diameter (cm) | Local ecotype | Opuntia dillenii | ,7286^*^ | ,05479 | ,000 | ,5639 | ,8933 |
|  |  | Opuntia robusta | -,8842^*^ | ,10527 | ,001 | -1,2490 | -,5194 |
|  | Opuntia dillenii | Local ecotype | -,7286^*^ | ,05479 | ,000 | -,8933 | -,5639 |
|  |  | Opuntia robusta | -1,6128^*^ | ,10573 | ,000 | -1,9770 | -1,2486 |
|  | Opuntia robusta | Local ecotype | ,8842^*^ | ,10527 | ,001 | ,5194 | 1,2490 |
|  |  | Opuntia dillenii | 1,6128^*^ | ,10573 | ,000 | 1,2486 | 1,9770 |
| Peel thickness center(cm) | Local ecotype | Opuntia dillenii | -,0298 | ,01852 | ,390 | -,0875 | ,0279 |
|  |  | Opuntia robusta | -,1118 | ,06829 | ,428 | -,3664 | ,1428 |
|  | Opuntia dillenii | Local ecotype | ,0298 | ,01852 | ,390 | -,0279 | ,0875 |
|  |  | Opuntia robusta | -,0820 | ,06732 | ,638 | -,3404 | ,1764 |
|  | Opuntia robusta | Local ecotype | ,1118 | ,06829 | ,428 | -,1428 | ,3664 |
|  |  | Opuntia dillenii | ,0820 | ,06732 | ,638 | -,1764 | ,3404 |
| Peel thickness base (cm) | Local ecotype | Opuntia dillenii | ,5954^*^ | ,08266 | ,003 | ,3002 | ,8906 |
|  |  | Opuntia robusta | ,2646 | ,08227 | ,074 | -,0315 | ,5607 |
|  | Opuntia dillenii | Local ecotype | -,5954^*^ | ,08266 | ,003 | -,8906 | -,3002 |
|  |  | Opuntia robusta | -,3308^*^ | ,03526 | ,000 | -,4368 | -,2248 |
|  | Opuntia robusta | Local ecotype | -,2646 | ,08227 | ,074 | -,5607 | ,0315 |
|  |  | Opuntia dillenii | ,3308^*^ | ,03526 | ,000 | ,2248 | ,4368 |
| Fruit weight (g) | Local ecotype | Opuntia dillenii | 39,7760^*^ | 3,79125 | ,001 | 25,1913 | 54,3607 |
|  |  | Opuntia robusta | -26,5340 | 11,17482 | ,179 | -65,8500 | 12,7820 |
|  | Opuntia dillenii | Local ecotype | -39,7760^*^ | 3,79125 | ,001 | -54,3607 | -25,1913 |
|  |  | Opuntia robusta | -66,3100^*^ | 10,54097 | ,010 | -107,7148 | -24,9052 |
|  | Opuntia robusta | Local ecotype | 26,5340 | 11,17482 | ,179 | -12,7820 | 65,8500 |
|  |  | Opuntia dillenii | 66,3100^*^ | 10,54097 | ,010 | 24,9052 | 107,7148 |
| Peel weight (g) | Local ecotype | Opuntia dillenii | 18,1520^*^ | 1,72815 | ,001 | 11,4035 | 24,9005 |
|  |  | Opuntia robusta | -15,7560 | 5,24274 | ,088 | -34,2786 | 2,7666 |
|  | Opuntia dillenii | Local ecotype | -18,1520^*^ | 1,72815 | ,001 | -24,9005 | -11,4035 |
|  |  | Opuntia robusta | -33,9080^*^ | 4,95445 | ,007 | -53,4094 | -14,4066 |
|  | Opuntia robusta | Local ecotype | 15,7560 | 5,24274 | ,088 | -2,7666 | 34,2786 |
|  |  | Opuntia dillenii | 33,9080^*^ | 4,95445 | ,007 | 14,4066 | 53,4094 |
| Pulp weight (g) | Local ecotype | Opuntia dillenii | 21,3340^*^ | 2,40449 | ,002 | 12,2063 | 30,4617 |
|  |  | Opuntia robusta | -11,2240 | 9,66538 | ,662 | -46,9296 | 24,4816 |
|  | Opuntia dillenii | Local ecotype | -21,3340^*^ | 2,40449 | ,002 | -30,4617 | -12,2063 |
|  |  | Opuntia robusta | -32,5580 | 9,38214 | ,074 | -69,4342 | 4,3182 |
|  | Opuntia robusta | Local ecotype | 11,2240 | 9,66538 | ,662 | -24,4816 | 46,9296 |
|  |  | Opuntia dillenii | 32,5580 | 9,38214 | ,074 | -4,3182 | 69,4342 |
| Ratio weight peep/fruit | Local ecotype | Opuntia dillenii | -,7895 | 1,72231 | ,961 | -6,0642 | 4,4852 |
|  |  | Opuntia robusta | -6,5497 | 7,13044 | ,791 | -33,5092 | 20,4099 |
|  | Opuntia dillenii | Local ecotype | ,7895 | 1,72231 | ,961 | -4,4852 | 6,0642 |
|  |  | Opuntia robusta | -5,7602 | 7,07293 | ,842 | -32,9603 | 21,4399 |
|  | Opuntia robusta | Local ecotype | 6,5497 | 7,13044 | ,791 | -20,4099 | 33,5092 |
|  |  | Opuntia dillenii | 5,7602 | 7,07293 | ,842 | -21,4399 | 32,9603 |
| Ratio weight pulp/fruit | Local ecotype | Opuntia dillenii | ,4278 | 1,69158 | ,993 | -4,8063 | 5,6620 |
|  |  | Opuntia robusta | 5,8362 | 7,22314 | ,844 | -21,4863 | 33,1588 |
|  | Opuntia dillenii | Local ecotype | -,4278 | 1,69158 | ,993 | -5,6620 | 4,8063 |
|  |  | Opuntia robusta | 5,4084 | 7,15529 | ,868 | -22,2020 | 33,0188 |
|  | Opuntia robusta | Local ecotype | -5,8362 | 7,22314 | ,844 | -33,1588 | 21,4863 |
|  |  | Opuntia dillenii | -5,4084 | 7,15529 | ,868 | -33,0188 | 22,2020 |
| Weight of seeds/fruit | Local ecotype | Opuntia dillenii | -,7300^*^ | ,14791 | ,010 | -1,2307 | -,2293 |
|  |  | Opuntia robusta | -,7380^*^ | ,06800 | ,000 | -,9575 | -,5185 |
|  | Opuntia dillenii | Local ecotype | ,7300^*^ | ,14791 | ,010 | ,2293 | 1,2307 |
|  |  | Opuntia robusta | -,0080 | ,13941 | 1,000 | -,5249 | ,5089 |
|  | Opuntia robusta | Local ecotype | ,7380^*^ | ,06800 | ,000 | ,5185 | ,9575 |
|  |  | Opuntia dillenii | ,0080 | ,13941 | 1,000 | -,5089 | ,5249 |
| number of fully developed seeds | Local ecotype | Opuntia dillenii | 58,2000^*^ | 9,96092 | ,001 | 27,8675 | 88,5325 |
|  |  | Opuntia robusta | -63,4000^*^ | 9,39468 | ,001 | -92,7007 | -34,0993 |
|  | Opuntia dillenii | Local ecotype | -58,2000^*^ | 9,96092 | ,001 | -88,5325 | -27,8675 |
|  |  | Opuntia robusta | -121,6000^*^ | 8,08455 | ,000 | -146,0630 | -97,1370 |
|  | Opuntia robusta | Local ecotype | 63,4000^*^ | 9,39468 | ,001 | 34,0993 | 92,7007 |
|  |  | Opuntia dillenii | 121,6000^*^ | 8,08455 | ,000 | 97,1370 | 146,0630 |
| number of abortive seeds | Local ecotype | Opuntia dillenii | 193,8000^*^ | 10,32666 | ,000 | 153,1254 | 234,4746 |
|  |  | Opuntia robusta | 190,0000^*^ | 10,37786 | ,000 | 149,5836 | 230,4164 |
|  | Opuntia dillenii | Local ecotype | -193,8000^*^ | 10,32666 | ,000 | -234,4746 | -153,1254 |
|  |  | Opuntia robusta | -3,8000 | 1,06771 | ,062 | -7,8460 | ,2460 |
|  | Opuntia robusta | Local ecotype | -190,0000^*^ | 10,37786 | ,000 | -230,4164 | -149,5836 |
|  |  | Opuntia dillenii | 3,8000 | 1,06771 | ,062 | -,2460 | 7,8460 |
| Based on observed means.  The error term is Mean Square(Error) = 179,567. | | | | | | | |
| *. The mean difference is significant at the ,05 level. | | | | | | | |
